# Supplementary material for: Audience segmentation of New Zealand cat owners: Understanding the barriers and drivers of cat containment behavior
Source: PLoS One. 2024 Jan 10;19(1):e0296805. doi: 10.1371/journal.pone.0296805 (PMC10781190; doi:10.1371/journal.pone.0296805)
Supplement: S1 Appendix — (PDF) [file pone.0296805.s001.pdf]

Cat containment questionnaire

Survey Flow: Block Randomiser

1. Randomise order of Allow Roaming and Prevent Roaming Blocks and Concern and Beliefs Block.
2. Randomise order to Allow Roaming Block and Prevent Roaming Block.

Start of Block: Captcha

Q1.1 Before you proceed with the survey, please complete the captcha below

End of Block: Captcha

Start of Block: Informed Consent

Q2.1
Welcome to the research study!

We are interested in understanding cat owners' behaviour and views towards cat management. Your participation in this study will assist in the development of cat management programmes to protect native wildlife and the welfare of owned cats in New Zealand. You will be asked to answer some questions about yourself, your cat, and your views towards cat ownership practices. Your responses will be kept completely confidential.

The questionnaire should take you around 10-15 minutes to complete. Your participation in this research is voluntary. You have the right to withdraw at any point during the study. The Principal Investigator of this study is Sarah Chamberlain and can be contacted at sch427@uclive.ac.nz.

This project has been approved by the Educational Research Human Ethics Committee. This questionnaire is unlikely to raise any personal or upsetting issues, but if it does you may wish to contact Lifeline on 0800 543 354 or your GP.

By clicking the button below, you acknowledge:

Your participation in this research is voluntary.
You are 18 years of age or over
You agree to participate in this study and are aware that you may choose to withdraw at any time for any reason.

End of Block: Informed Consent

Start of Block: Screening & Demographics

Q3.1 **These first questions are to find out something about you**

Q3.2_gender What is your gender?

- Male
- Female
- Non-binary / third gender
- Prefer not to say

Q3.3_locality Which of the following best describes the area where you live?

- Inner city
- Suburban
- Residential in rural town
- Semi-rural / Peri-urban (e.g., acreage on the edge of town)
- Rural

Q3.4_ethnicity What ethnic group do you belong to?
Select all that apply to you.

- [ ] New Zealand European / Pākehā
- [ ] Māori
- [ ] Samoan
- [ ] Cook Islands Māori
- [ ] Tongan
- [ ] Niuean
- [ ] Chinese
- [ ] Indian
- [ ] Other (please specify) ____

Q3.5_education What is the highest qualification you have received?

- ○ Less than secondary school
- ○ Secondary school qualification
- ○ Trade / technical certificate or professional qualification
- ○ Undergraduate qualification (Bachelors degree or Undergraduate Diploma)
- ○ Bachelor Honours or Postgraduate Certificate, Diploma or Degree
- ○ Masters degree
- ○ PhD

Q3.6_home garden In what kind of dwelling do you currently live?

- ○ House with large / medium outside space (e.g., large or medium garden)
- ○ House with small outside space (e.g., patio or small garden / courtyard)
- ○ Semi-detached, terrace, or townhouse with medium space outside (e.g., medium garden)
- ○ Semi-detached, terrace, or townhouse with small outside space (e.g., patio or small garden / courtyard)
- ○ Flat, unit, apartment with small outside space (e.g., balcony or patio)
- ○ Flat, unit, apartment with no outside space
- ○ Other (please specify) ____

Q3.7_home ownership Which of the following best describes your ownership of your home?

- ○ I (or my family) own it
- ○ I (or my family) rent it
- ○ Other (please specify) ____

Q3.8_year of birth What is your year of birth?

____

Q3.9_cat ownership Do you currently own a cat?

- ◯ Yes I own a cat
- ◯ I have previously owned a cat but not currently
- ◯ No but a cat likes to visit
- ◯ No I do not own a cat

End of Block: Screening & Demographics

Start of Block: Cat demographics

Q4.1 **These questions are about your cat.**

X→

Q4.2_no. cats owned
How many cats do you currently own?

▼ none ... 20 or more

X→ X→

Q4.3_desexed Is your cat desexed?
Select one of the following.

- ◯ All of my cats are desexed
- ◯ Some of my cats are desexed
- ◯ My cats are not desexed
- ◯ Unsure

Q4.4_microchipped Is your cat microchipped?
Select one of the following.

- All of my cats are microchipped
- Some of my cats are microchipped
- My cats are not microchipped
- Unsure

Q4.5_collar ID
Does your cat wear a collar with an ID tag (phone number / address)?
Select one of the following.

- All of my cats wear an ID collar
- Some of my cats wear an ID collar
- My cats do not wear an ID collar

Q4.6_collar bell
Does your cat wear a collar with a bell?
Select one of the following.

- All of my cats wear a collar with a bell
- Some of my cats wear a collar with a bell
- My cats do not wear a collar with a bell

Q4.7_awareness How often do you think your cat kills wildlife when outside? (e.g., birds, lizards, rodents, or other small mammals)

- ◯ Never
- ◯ Rarely
- ◯ Sometimes
- ◯ Often
- ◯ Always

Q4.8_awareness How much do you agree with the following statement?
"In a typical day, my cat does not roam far from my house"

- ◯ Strongly disagree
- ◯ Disagree
- ◯ Neither agree nor disagree
- ◯ Agree
- ◯ Strongly agree

Q4.9_awareness How important do you think it is for owners to provide the following for their cat?

	Not at all important	Slightly important	Somewhat important	Very important	Extremely important
Food & water	○	○	○	○	○
Bedding	○	○	○	○	○
Scratching post	○	○	○	○	○
Access to fresh air (e.g., slightly opened window)	○	○	○	○	○
Places to hide	○	○	○	○	○
Vantage points / windows to look out from	○	○	○	○	○
Toys	○	○	○	○	○
Food puzzles	○	○	○	○	○
Companionship	○	○	○	○	○

Q4.10_Knowledge What comes to mind when you hear the phrase 'cat containment'? Select all that apply.

- ▢ Keeping cats indoors all the time
- ▢ Keeping cats indoors only at night
- ▢ Allowing cats outdoor access for only short periods
- ▢ Allowing cats outdoors only when fully supervised
- ▢ Allowing cats outdoor access in a yard with a solid timber or metal fence
- ▢ Allowing cats outdoor access only in a cat enclosure / run, specially constructed cat-escape proof yard, or while on a harness and lead
- ▢ Other ____

Q4.11_emotionalreact How would you describe your feelings about the phrase 'cat containment'?

- ○ Very negative
- ○ Negative
- ○ Neither positive nor negative
- ○ Positive
- ○ Very positive

End of Block: Cat demographics

Start of Block: Behaviour / Intentions

Q5.1 **Please read the following definitions before completing the next questions.**

Free roaming cats: cats that are allowed to leave the house and the owner's property.

Cat containment: cats can be contained to prevent them from roaming freely. This includes keeping them indoors full-time or overnight, or when they are outside, keeping them in a cat escape-proof fenced yard or enclosure, on a harness and lead, or fully supervised.

Cat escape-proof fence: specially constructed fence that cats cannot jump or climb over and prevents cats from leaving the backyard or property. Standard backyard fences are NOT cat-proof.

Cat enclosure: an enclosed area (such as a purpose-built run or netted balcony area) where cats can access the outdoors but cannot roam freely.

Q5.2_contain behavio
How often do you do each of the following?

	Never	Rarely	Sometimes	Often	Always
Allow your cat to roam freely when outside	○	○	○	○	○
Keep your cat indoors	○	○	○	○	○
Keep your cat indoors overnight	○	○	○	○	○
Confine your cat to an escape-proof fenced yard when outside	○	○	○	○	○
Confine your cat to an enclosure when outside	○	○	○	○	○
Walk your cat on a harness and lead when outside	○	○	○	○	○
Fully supervise your cat when outside (e.g., cat is allowed to explore but is always within eyesight)	○	○	○	○	○

Q5.3_contain intent

How often do you expect to do each of the following behaviours during the next 6 months?

	Never	Rarely	Sometimes	Often	Always
Allow your cat to roam freely when outside	○	○	○	○	○
Keep your cat indoors	○	○	○	○	○
Keep your cat indoors overnight	○	○	○	○	○
Confine your cat to an escape-proof fenced yard when outside	○	○	○	○	○
Confine your cat to an enclosure when outside	○	○	○	○	○
Walk your cat on a harness and lead when outside	○	○	○	○	○
Fully supervise your cat when outside	○	○	○	○	○

End of Block: Behaviour / Intentions

Start of Block: COM-B 2 allow roaming

Q6.1

How much do you agree or disagree with the following reasons why cats should be **allowed to roam freely?**

X→

Q6.2_motivation roam *Cats do not like being contained*

- Strongly disagree
- Disagree
- Neither agree nor disagree
- Agree
- Strongly agree

Q6.3_enviro context *My home is too small to keep my cat contained*

- Strongly disagree
- Disagree
- Neither agree nor disagree
- Agree
- Strongly agree

Q6.4_motivation roam *My cat destroys furniture if it doesn't roam freely*

- Strongly disagree
- Disagree
- Neither agree nor disagree
- Agree
- Strongly agree

Q6.5_motivation roam *My cat should be allowed to roam freely as I don't want to have a litter box in my home*

- Strongly disagree
- Disagree
- Neither agree nor disagree
- Agree
- Strongly agree

Q6.6_physical *My cat howls if it can't get outside*

- Strongly disagree
- Disagree
- Neither agree nor disagree
- Agree
- Strongly agree

Q6.7_affordability *It is too expensive to buy kitty litter*

- Strongly disagree
- Disagree
- Neither agree nor disagree
- Agree
- Strongly agree

Q6.8_enviro context *Containing my cat is difficult in my current residential circumstances*

- Strongly disagree
- Disagree
- Neither agree nor disagree
- Agree
- Strongly agree

Q6.9_enviro context *I am not allowed to make changes to my home or property to keep my cat contained*

- Strongly disagree
- Disagree
- Neither agree nor disagree
- Agree
- Strongly agree

Q6.10_physical *I find it difficult to contain my cat as kids or visitors sometimes leave the door open*

- Strongly disagree
- Disagree
- Neither agree nor disagree
- Agree
- Strongly agree

Q6.11_Knowledge *I don't know how to make changes to my home or property to keep my cat contained*

- Strongly disagree
- Disagree
- Neither agree nor disagree
- Agree
- Strongly agree

Q6.12_motivationroam *Allowing cats to roam freely is good for their physical health*

- Strongly disagree
- Disagree
- Neither agree nor disagree
- Agree
- Strongly agree

Q6.13_affordability *It is too expensive to make changes to my home or property to keep my cat contained*

- Strongly disagree
- Disagree
- Neither agree nor disagree
- Agree
- Strongly agree

Q6.14_attention A *To show that you have read this sentence, in this line we ask you to mark 'agree' on the answer scale*

- Strongly disagree
- Disagree
- Neither agree nor disagree
- Agree
- Strongly agree

Q6.15_motivationroam *Allowing cats to roam freely is good for their mental health*

- Strongly disagree
- Disagree
- Neither agree nor disagree
- Agree
- Strongly agree

Q6.16_motivationroam *I don't want to make changes to my home or property to keep my cat contained*

- Strongly disagree
- Disagree
- Neither agree nor disagree
- Agree
- Strongly agree

Q6.17_motivationroam *Free roaming cats are important for controlling rodents and pests*

- Strongly disagree
- Disagree
- Neither agree nor disagree
- Agree
- Strongly agree

Q6.18_motivationroam *Cats that wear collars with a bell do not need to be contained because they are less likely to kill wildlife*

- Strongly disagree
- Disagree
- Neither agree nor disagree
- Agree
- Strongly agree

Q6.19_Socialrole *Cats should be allowed to roam freely as owners should not be responsible for managing their cats hunting*

- Strongly disagree
- Disagree
- Neither agree nor disagree
- Agree
- Strongly agree

Q6.20_perceiv effort *Cats should be allowed to roam freely as it is too difficult to contain them*

- Strongly disagree
- Disagree
- Neither agree nor disagree
- Agree
- Strongly agree

X→

Q6.21_emotionreact *I would feel guilty if I didn't let my cat roam freely*

- Strongly disagree
- Disagree
- Neither agree nor disagree
- Agree
- Strongly agree

X→

Q6.22_memoryattentio *I find it difficult to contain my cat because I sometimes forget to keep the doors and windows shut*

- Strongly disagree
- Disagree
- Neither agree nor disagree
- Agree
- Strongly agree

Q6.23_habit *My cat should not be contained as it has always been allowed to roam freely*

- Strongly disagree
- Disagree
- Neither agree nor disagree
- Agree
- Strongly agree

Q6.24_time *I don't have time to make changes to my home or property to keep my cat contained*

- Strongly disagree
- Disagree
- Neither agree nor disagree
- Agree
- Strongly agree

Q6.25_resourceavail *I don't have access to the materials needed to make changes to my home or property to keep my cat contained.*

- Strongly disagree
- Disagree
- Neither agree nor disagree
- Agree
- Strongly agree

Q6.26_envirocontext *My neighbours don't want to have a cat escape-proof fence*

- Strongly disagree
- Disagree
- Neither agree nor disagree
- Agree
- Strongly agree

Q6.27_motivationroam *The benefits of letting my cat roam free outweigh the risks*

- Strongly disagree
- Disagree
- Neither agree nor disagree
- Agree
- Strongly agree

End of Block: COM-B 2 allow roaming

Start of Block: COM-B prevent roaming

Q7.1_interpersonal How much do you think the following people would **support cat containment?**

	Strongly oppose	Oppose	Neutral	Support	Strongly support
Other cat owners	○	○	○	○	○
My veterinarian	○	○	○	○	○
My friends and family	○	○	○	○	○
My neighbours	○	○	○	○	○

Q7.2
How much do you agree or disagree with the following statements?

	Strongly disagree	Disagree	Neither agree nor disagree	Agree	Strongly Agree
I am confident I can provide everything my cat needs to ensure he / she is happy when contained	○	○	○	○	○
I am confident I can keep my cat contained at all times	○	○	○	○	○
I would contain my cat if the local council made it compulsory	○	○	○	○	○
To show that you have read this sentence, in this line we ask you to mark 'Strongly disagree' on the answer scale	○	○	○	○	○
I would contain my cat if most other people in my community did so	○	○	○	○	○
I feel no moral obligation to contain my cat	○	○	○	○	○

Q7.3 How much do you agree or disagree with the following reasons why cats **should be contained?**

Q7.4_motivationconta *To keep cats safe*

- Strongly disagree
- Disagree
- Neither agree nor disagree
- Agree
- Strongly agree

Q7.5_motivationconta *To protect native wildlife*

- Strongly disagree
- Disagree
- Neither agree nor disagree
- Agree
- Strongly agree

Q7.6_emotionreaction *I would feel guilty if my cat was injured while roaming freely*

- Strongly disagree
- Disagree
- Neither agree nor disagree
- Agree
- Strongly agree

Q7.7_motivationconta *To prevent my cat from causing problems for the neighbours*

- ○ Strongly disagree
- ○ Disagree
- ○ Neither agree nor disagree
- ○ Agree
- ○ Strongly agree

Q7.8_motivationroam *Cats don't need to roam if they get enough enrichment (e.g., provided toys, things to scratch and climb, food puzzles, places to hide, access to fresh air and sunlight)*

- ○ Strongly disagree
- ○ Disagree
- ○ Neither agree nor disagree
- ○ Agree
- ○ Strongly agree

End of Block: COM-B prevent roaming

Start of Block: Concern & Beliefs

Q8.1_quality life
How do you think the following would impact a cat's quality of life?

	Very harmful	Harmful	Neither harmful nor beneficial	Beneficial	Very beneficial
Confining indoors overnight	○	○	○	○	○
Confining indoors at all times	○	○	○	○	○
Confining within a cat enclosure while outdoors	○	○	○	○	○
Confining within a cat escape-proof yard while outdoors	○	○	○	○	○

Q8.2_knowledge
How much do you agree or disagree with the following statements

	Strongly disagree	Disagree	Neither disagree nor agree	Agree	Strongly Agree
Pet cats pose a threat to native wildlife	○	○	○	○	○
Pet cats that wander off their owner's property are at risk of harm	○	○	○	○	○

Q8.3_concern roaming What is your level of concern about these **potential issues related to cats roaming freely?**

	Unconcerned	Slightly concerned	Somewhat concerned	Very concerned	Extremely concerned
Injury or death on the road	○	○	○	○	○
Getting lost	○	○	○	○	○
Killing wildlife	○	○	○	○	○
Conflict with other cats	○	○	○	○	○
Conflict with other animals (e.g., dogs, possums)	○	○	○	○	○
Causing problems for neighbours	○	○	○	○	○
To show that you have read this sentence, in this line we ask you to mark 'somewhat concerned' on the answer scale	○	○	○	○	○
Getting poisoned	○	○	○	○	○
Being stolen	○	○	○	○	○
Getting trapped	○	○	○	○	○
Catching a cat-specific disease (e.g., feline aids)	○	○	○	○	○
Catching a disease they can pass on to humans (e.g., toxoplasmosis)	○	○	○	○	○

End of Block: Concern & Beliefs
