# Supplementary material for: Audience segmentation of New Zealand cat owners: Understanding the barriers and drivers of cat containment behavior
Source: PLoS One. 2024 Jan 10;19(1):e0296805. doi: 10.1371/journal.pone.0296805 (PMC10781190; doi:10.1371/journal.pone.0296805)
Supplement: S1 Table — (DOCX) [file pone.0296805.s002.docx]

**Table S2. Capability, opportunity, and motivation variables and survey items**

| Components | Variables | Item Question |
| --- | --- | --- |
| Capability |  |  |
| Physical | Capability to Contain ^a^  (Cronbach’s α = 0.70) | How much do you agree or disagree with the following reasons why cats should be allowed to roam freely?  I find it difficult to contain my cat as kids or visitors sometimes leave the door open. ^r 1^ |
|  |  | My cat howls if it can't get outside. ^r 1^  My cat destroys furniture if it doesn’t roam. ^r 1^ |
| Memory |  | I find it difficult to contain my cat because I sometimes forget to keep the doors and windows shut ^r^ |
| Knowledge |  | I don't know how to make changes to my home or property to keep my cat contained ^r^ |
|  |  | How much do you agree with the following statements?  Pet cats that wander off their owner's property are at risk of being harmed. |
| Awareness |  | In a typical day, my cat does not roam far from my house.^3^ |
| Behavior   Regulation |  | I am confident that I can provide everything my cat needs to ensure he/she is happy when contained. ^3^  I am confident I can prevent my cat from roaming at all times. ^3^ |
| Opportunity |  |  |
| Time | Physical Opportunity to Contain ^b^ (Cronbach’s α = 0.81) | How much do you agree or disagree with the following statements?  I don't have the time to make changes to my home or property to keep my cat contained.^2^ |
| Resource   Availability |  | I don't have access to the materials needed to make changes to my home or property to keep my cat   contained. |
| Affordability |  | It is too expensive to make changes to my home or property to keep my cat contained. |
| Environmental   context |  | My home is too small to keep my cat contained.^1^  Containing my cat is difficult in my current residential circumstances.^3^ |
| Interpersonal   Influences | Social Opportunity to Contain ^c^  (Cronbach’s α = 0.86) | How much do you think the following people would support cat containment? ^3^  My veterinarian.  My neighbors.  My friends and family.  Other cat owners. |
| Motivation |  |  |
| Beliefs | Concern About Roaming ^d^  (Cronbach’s α = 0.95) | How concerned are you about the following potential roaming related issues? ^3^  Injury or death on the road.  Getting lost.  Killing wildlife.  Conflict with other cats.  Conflict with other animals (e.g., dogs, possums).  Causing problems for neighbors.  Getting poisoned.  Being stolen.  Getting trapped.  Catching a cat specific disease (e.g., feline aids).  Catching a disease they can pass onto humans (e.g., toxoplasmosis). |
|  | Containment is Beneficial for Cat Beliefs ^e^  (Cronbach’s α = 0.80) | How do you think the following would impact a cat's quality of life? ^2^  Confining indoors overnight.  Confining indoors at all times.  Confining within a cat enclosure while outdoors.  Confining within a cat escape-proof yard while outdoors. |
|  | Pro-Containment Beliefs ^a^  (Cronbach’s α = 0.90) | How much do you agree or disagree with the following reasons why cats should be contained? ^1234^  To keep cats safe. ^1^  To protect native wildlife. ^1^  To prevent my cat from causing problems for the neighbors. ^2^  Cats don’t need to roam if they get enough enrichment. ^4^ |
|  |  | How much do you agree or disagree with the following reasons why cats should be allowed to roam freely?  Cats do not like being contained. ^r 1^  My cat should be allowed to roam freely as I don’t want to have a litter box in my home. ^r 1^  Allowing cats to roam freely is good for their physical health. ^r 3^  Allowing cats to roam freely is good for their mental health. ^r 3^  I don't want to make changes to my home or property to keep my cat contained. ^r^  Roaming cats are important for controlling rodents & pests. ^r 2^  The benefits of roaming outweigh the risks. ^r 4^ |
| Perceived   Effort |  | Cats should be allowed to roam freely as owners should not be responsible for managing their cats hunting. ^r 4^ |
| Social Role   Identity |  | Cats should be allowed to roam freely as it is too difficult to contain them. ^r^ |
| Emotional   Reactions | Automatic  Motivation to Contain ^b^  (Cronbach’s α = 0.81) | How much do you agree or disagree with the following reasons why cats should be allowed to roam freely?  I would feel guilty if I didn’t let my cat roam freely. |
| Habit |  | My cat should not be contained as it has always been allowed to roam freely. |

Containment / contain refers to behaviors that cat owners can undertake to ensure their cat(s) remain on their property. Roaming refers to allowing cats to leave an owner’s property.

^r^ Reverse scored to represent drivers of containment.

^a^ All responses assessed on a five-point Likert scale (1=strongly disagree, 2=disagree, 3=neither agree nor disagree, 4=agree, 5=strongly agree).

^b^ All responses assessed on a five-point Likert scale (1=strongly agree, 2=agree, 3=neither agree nor disagree, 4=disagree, 5=strongly disagree).

^c^ All responses assessed on a five-point Likert scale (1=strongly oppose, 2=oppose, 3=neutral, 4=support, 5=strongly support).

^d^ All responses assessed on a five-point Likert scale (1=unconcerned, 2=slightly concerned, 3= somewhat concerned, 4=very concerned, 5=extremely concerned).

^e^ All responses assessed on a five-point Likert scale (1=very harmful, 2=harmful, 3=neither beneficial nor beneficial, 4=beneficial, 5=very beneficial).

^1^ McLeod et al. (2020), ^2^ McLeod et al. (2015a), ^3^ Elliott et al. (2019), ^4^ Crowley et al. (2020).
