# Supplementary material for: Audience segmentation of New Zealand cat owners: Understanding the barriers and drivers of cat containment behavior
Source: PLoS One. 2024 Jan 10;19(1):e0296805. doi: 10.1371/journal.pone.0296805 (PMC10781190; doi:10.1371/journal.pone.0296805)
Supplement: S2 Table — (DOCX) [file pone.0296805.s003.docx]

**Table S3. Summary of descriptive statistics for the four cat owner profiles for the capability, opportunity, and motivations (COM) factors and demographics**

| Variable | Engaged  (*n* = 22) | | Receptive  (*n* = 68) | | Ambivalent  (*n* = 188) | | Opposed  (*n* = 117) | |
| --- | --- | --- | --- | --- | --- | --- | --- | --- |
|  | *M* | *SD* | *M* | *SD* | *M* | *SD* | *M* | *SD* |
| COM factors |  |  |  |  |  |  |  |  |
| Capability to Contain | 4.54 | 0.40 | 3.45 | 0.45 | 3.06 | 0.49 | 2.78 | 0.56 |
| Physical Opportunity to Contain | 4.33 | 0.74 | 3.18 | 0.70 | 2.82 | 0.76 | 2.48 | 0.96 |
| Social Opportunity to Contain | 3.95 | 0.56 | 3.44 | 0.53 | 2.74 | 0.64 | 1.94 | 0.64 |
| Concern About Roaming | 3.94 | 0.93 | 3.41 | 0.91 | 2.85 | 0.99 | 2.07 | 0.76 |
| Containment is Beneficial for   Cat Beliefs | 4.07 | 0.60 | 3.28 | 0.48 | 2.64 | 0.49 | 1.83 | 0.49 |
| Pro-Containment Beliefs | 4.27 | 0.42 | 3.32 | 0.33 | 2.59 | 0.32 | 1.85 | 0.31 |
| Automatic Motivation to   Contain | 4.63 | 0.41 | 3.09 | 0.62 | 1.92 | 0.59 | 1.23 | 0.41 |
| Year of Birth | 1974 | 17.20 | 1978 | 16.43 | 1974 | 17.28 | 1972 | 17.22 |
| No. cats owned | 1.59 | 0.96 | 1.38 | 0.62 | 1.62 | 1.05 | 1.5 | 0.75 |
|  | *n* | *%* | *n* | *%* | *n* | *%* | *n* | *%* |
| Gender |  |  |  |  |  |  |  |  |
| Male | 8 | 36.4 | 22 | 32.4 | 62 | 33.00 | 45 | 38.2 |
| Female | 14 | 63.6 | 46 | 67.6 | 123 | 65.40 | 71 | 60.7 |
| Other | - | - | - | - | 3 | 1.60 | 1 | 0.09 |
| Locality |  |  |  |  |  |  |  |  |
| Urban | 5 | 22.7 | 6 | 8.8 | 13 | 6.90 | 8 | 6.9 |
| Suburban | 10 | 45.5 | 47 | 69.1 | 125 | 66.50 | 68 | 58.1 |
| Rural Residential | 6 | 27.3 | 7 | 10.3 | 29 | 15.40 | 19 | 16.2 |
| Semi-Rural | - | - | 2 | 2.9 | 9 | 4.80 | 11 | 9.4 |
| Rural | 1 | 4.5 | 6 | 8.8 | 12 | 6.40 | 11 | 9.4 |
| Ethnicity |  |  |  |  |  |  |  |  |
| NZ European /   Pākehā | 19 | 86.4 | 51 | 75 | 160 | 85.10 | 17 | 85.5 |
| Māori | 2 | 9.1 | 10 | 14.7 | 19 | 10.10 | 10 | 8.5 |
| Pacifica | - | - | 1 | 5.9 | 9 | 4.8 | 2 | 1.8 |
|  | *n* | *%* | *n* | *%* | *n* | *%* | *n* | *%* |
| Chinese | 1 | 4.5 | 2 | 2.9 | 2 | 1.10 | 3 | 2.6 |
| Indian | - | - | 3 | 4.4 | 1 | 0.5 | 2 | 1.7 |
| Other | 2 | 9.1 | 10 | 14.7 | 12 | 6.40 | 8 | 6.8 |
| Highest Education level |  |  |  |  |  |  |  |  |
| None | 1 | 4.5 | 7 | 10.3 | 8 | 4.30 | 8 | 6.8 |
| Secondary School | 6 | 27.3 | 13 | 19.1 | 61 | 32.40 | 33 | 28.2 |
| Trade / Technical | 4 | 18.2 | 17 | 25.0 | 53 | 28.20 | 35 | 29.9 |
| Undergraduate | 3 | 13.6 | 14 | 20.6 | 30 | 16.00 | 20 | 17.1 |
| Postgraduate | 4 | 18.2 | 13 | 19.1 | 24 | 12.80 | 16 | 13.7 |
| Masters | 2 | 9.1 | 2 | 2.9 | 11 | 5.90 | 4 | 3.4 |
| PhD | 2 | 9.1 | 2 | 2.9 | 1 | 0.50 | 1 | 0.9 |
| Dwelling type |  |  |  |  |  |  |  |  |
| House large garden | 11 | 50 | 33 | 48.5 | 130 | 69.10 | 77 | 65.8 |
| House medium garden | 5 | 22.7 | 14 | 20.6 | 31 | 16.50 | 24 | 20.5 |
| Townhouse medium garden | 1 | 4.5 | 5 | 7.4 | 8 | 4.30 | 2 | 1.7 |
| Townhouse small garden | 1 | 4.5 | 6 | 8.8 | 5 | 2.70 | 5 | 4.3 |
| Flat, unit or apartment with   small outdoor space | 2 | 9.1 | 9 | 13.2 | 8 | 4.30 | 5 | 4.3 |
| Flat, unit or apartment no   outdoor space | 1 | 4.5 | 1 | 1.5 | 1 | 0.50 | - | - |
| Other | 1 | 4.5 | - | - | 5 | 2.70 | 4 | 3.4 |
| Home ownership |  |  |  |  |  |  |  |  |
| Own | 15 | 68.2 | 35 | 51.5 | 131 | 69.70 | 76 | 65.0 |
| Rent | 7 | 31.8 | 32 | 47.1 | 53 | 28.20 | 38 | 32.5 |
| Other | - | - | 1 | 1.5 | 4 | 2.10 | 3 | 2.6 |

All COM variables range from a minimum 1 to maximum 5 and are scored to reflect drivers of containment. Containment / contain refers to behaviors that cat owners can undertake to ensure their cat(s) remain on their property. Roaming refers to allowing cats to leave an owner’s property.
