# Supplementary material for: Audience segmentation of New Zealand cat owners: Understanding the barriers and drivers of cat containment behavior
Source: PLoS One. 2024 Jan 10;19(1):e0296805. doi: 10.1371/journal.pone.0296805 (PMC10781190; doi:10.1371/journal.pone.0296805)
Supplement: Attachment — Submitted filename: PONE-D-23-20541_reviewer comments.pdf [file pone.0296805.s004.pdf]

PLOS ONE

Audience segmentation of New Zealand cat owners: Understanding the barriers and drivers of cat containment behavior

--Manuscript Draft--

**Manuscript Number:**	PONE-D-23-20541
**Article Type:**	Research Article
**Full Title:**	Audience segmentation of New Zealand cat owners: Understanding the barriers and drivers of cat containment behavior
**Short Title:**	Barriers and drivers of cat containment
**Corresponding Author:**	Sarah A. E. Chamberlain<br>University of Canterbury<br>Christchurch, NEW ZEALAND
**Keywords:**	domestic cat management; human behavior change; behavior change wheel; intervention design; audience segmentation
**Abstract:**	Free-roaming companion cats have a detrimental impact on the environment and are at risk of harm. Despite these negative impacts, it is the norm in New Zealand (NZ) to allow companion cats to roam freely and only a minority of cat owners practice cat containment.<br>This study, using audience segmentation analysis, examined the main barriers and drivers of participation in cat containment for NZ cat owners. A quantitative online cross-sectional survey of 395 NZ cat owners was conducted, measuring containment intentions and behavior, and capability, opportunity, and motivation (COM) to perform cat containment. Results from bivariate correlations and multiple regression demonstrated that the COM factors predicted increased cat containment intentions and behavior. Latent profile analysis identified four distinct segments of cat owners with unique COM profiles; engaged (6%), receptive (17%), ambivalent (48%), and opposed (30%). Validation analysis demonstrated that these groups all differed significantly in their cat containment intentions and behaviors. From these findings theoretically grounded behavior change interventions can be developed to target the causes of non-participation in cat containment for each of the identified cat owner segments, thereby improving the management of free-roaming cats in NZ.
**Order of Authors:**	Sarah A. E. Chamberlain
	Lynette J. McLeod
	Donald W. Hine
**Additional Information:**	
**Question**	**Response**
**Financial Disclosure**<br><br>Enter a financial disclosure statement that describes the sources of funding for the work included in this submission. Review the submission guidelines for detailed requirements. View published research articles from *PLOS ONE* for specific examples.<br><br>This statement is required for submission and **will appear in the published article** if the submission is accepted. Please make sure it is accurate.	The authors received no specific funding for this work.

Powered by Editorial Manager® and ProduXion Manager® from Aries Systems Corporation

**Unfunded studies**<br>Enter: *The author(s) received no specific funding for this work.*<br><br>**Funded studies**<br>Enter a statement with the following details:<br>• Initials of the authors who received each award<br>• Grant numbers awarded to each author<br>• The full name of each funder<br>• URL of each funder website<br>• Did the sponsors or funders play any role in the study design, data collection and analysis, decision to publish, or preparation of the manuscript?<br>• **NO** - Include this sentence at the end of your statement: *The funders had no role in study design, data collection and analysis, decision to publish, or preparation of the manuscript.*<br>• **YES** - Specify the role(s) played.<br><br>* typeset	
**Competing Interests**<br><br>Use the instructions below to enter a competing interest statement for this submission. On behalf of all authors, disclose any competing interests that could be perceived to bias this work—acknowledging all financial support and any other relevant financial or non-financial competing interests.<br><br>This statement is required for submission and **will appear in the published article** if the submission is accepted. Please make sure it is accurate and that any funding sources listed in your Funding Information later in the submission form are also declared in your Financial Disclosure statement.<br><br>View published research articles from *PLOS ONE* for specific examples.	The authors have declared that no competing interests exist.

**NO authors have competing interests**<br><br>Enter: *The authors have declared that no competing interests exist.*<br><br>**Authors with competing interests**<br><br>Enter competing interest details beginning with this statement:<br><br>*I have read the journal's policy and the authors of this manuscript have the following competing interests: [insert competing interests here]*<br><br>* typeset	
**Ethics Statement**<br><br>Enter an ethics statement for this submission. This statement is required if the study involved:<br><br>• Human participants<br>• Human specimens or tissue<br>• Vertebrate animals or cephalopods<br>• Vertebrate embryos or tissues<br>• Field research<br><br>Write "N/A" if the submission does not require an ethics statement.<br><br>General guidance is provided below. Consult the submission guidelines for detailed instructions. **Make sure that all information entered here is included in the Methods section of the manuscript.**	The study received blanket ethics approval from the Human Ethics Committee of the University of Canterbury.<br><br>Participants' informed written consent was obtained.

Powered by Editorial Manager® and ProduXion Manager® from Aries Systems Corporation

**Format for specific study types**<br><br>**Human Subject Research (involving human participants and/or tissue)**<br>• Give the name of the institutional review board or ethics committee that approved the study<br>• Include the approval number and/or a statement indicating approval of this research<br>• Indicate the form of consent obtained (written/oral) or the reason that consent was not obtained (e.g. the data were analyzed anonymously)<br><br>**Animal Research (involving vertebrate animals, embryos or tissues)**<br>• Provide the name of the Institutional Animal Care and Use Committee (IACUC) or other relevant ethics board that reviewed the study protocol, and indicate whether they approved this research or granted a formal waiver of ethical approval<br>• Include an approval number if one was obtained<br>• If the study involved *non-human primates*, add *additional details* about animal welfare and steps taken to ameliorate suffering<br>• If anesthesia, euthanasia, or any kind of animal sacrifice is part of the study, include briefly which substances and/or methods were applied<br><br>**Field Research**<br><br>Include the following details if this study involves the collection of plant, animal, or other materials from a natural setting:<br>• Field permit number<br>• Name of the institution or relevant body that granted permission	
**Data Availability**<br><br>Authors are required to make all data underlying the findings described fully available, without restriction, and from the time of publication. PLOS allows rare exceptions to address legal and ethical concerns. See the PLOS Data Policy and FAQ for detailed information.	Yes - all data are fully available without restriction

Powered by Editorial Manager® and ProduXion Manager® from Aries Systems Corporation

A Data Availability Statement describing where the data can be found is required at submission. Your answers to this question constitute the Data Availability Statement and **will be published in the article**, if accepted.<br><br>**Important:** Stating 'data available on request from the author' is not sufficient. If your data are only available upon request, select 'No' for the first question and explain your exceptional situation in the text box.<br><br>Do the authors confirm that all data underlying the findings described in their manuscript are fully available without restriction?	
**Describe where the data may be found in full sentences. If you are copying our sample text, replace any instances of XXX with the appropriate details.**<br><br>• If the data are **held or will be held in a public repository**, include URLs, accession numbers or DOIs. If this information will only be available after acceptance, indicate this by ticking the box below. For example: *All XXX files are available from the XXX database (accession number(s) XXX, XXX.).*<br>• If the data are all contained **within the manuscript and/or Supporting Information files**, enter the following: *All relevant data are within the manuscript and its Supporting Information files.*<br>• If neither of these applies but you are able to provide **details of access elsewhere**, with or without limitations, please do so. For example:<br><br>*Data cannot be shared publicly because of [XXX]. Data are available from the XXX Institutional Data Access / Ethics Committee (contact via XXX) for researchers who meet the criteria for access to confidential data.*<br><br>*The data underlying the results presented in the study are available from (include the name of the third party*	The data file is available from the Mendeley database (DOI: 10.17632/hytxh9bm8c.1)

and contact information or URL).<br>• This text is appropriate if the data are owned by a third party and authors do not have permission to share the data.<br><br>* typeset	
Additional data availability information:	

Manuscript

Click here to access/download;Manuscript;Barriers and Drivers of Cat Containment_manuscript_10.7.23.docx

Audience segmentation of New Zealand cat owners: Understanding the barriers and drivers of cat containment behavior

Sarah A. E. Chamberlain[1], Lynette J. McLeod[1] and Donald W. Hine[1*]

[1]School of Psychology, Speech, and Hearing, University of Canterbury, Christchurch, New Zealand

Sarah A. E. Chamberlain https://orcid.org/0000-0002-0060-8046

Lynette J. McLeod https://orcid.org/0000-0001-9408-3342

Donald W. Hine https://orcid.org/0000-0002-3905-7026

We have no conflicts of interest to disclose.

* Corresponding author

E-mail: donald.hine@canterbury.ac.nz (DWH)

Abstract

Free-roaming companion cats have a detrimental impact on the environment and are at risk of harm. Despite these negative impacts, it is the norm in New Zealand (NZ) to allow companion cats to roam freely and only a minority of cat owners practice cat containment. This study, using audience segmentation analysis, examined the main barriers and drivers of participation in cat containment for NZ cat owners. A quantitative online cross-sectional survey of 395 NZ cat owners was conducted, measuring containment intentions and behavior, and capability, opportunity, and motivation (COM) to perform cat containment. Results from bivariate correlations and multiple regression demonstrated that the COM factors predicted increased cat containment intentions and behavior. Latent profile analysis identified four distinct segments of cat owners with unique COM profiles; *engaged* (6%), *receptive* (17%), *ambivalent* (48%), and *opposed* (30%). Validation analysis demonstrated that these groups all differed significantly in their cat containment intentions and behaviors. From these findings theoretically grounded behavior change interventions can be developed to target the causes of non-participation in cat containment for each of the identified cat owner segments, thereby improving the management of free-roaming cats in NZ.

Keywords: domestic cat management, human behavior change, behavior change wheel, intervention design, audience segmentation

Introduction

The cat is New Zealand's (NZ) most popular companion animal with an estimated population of 1.2 million [1, 2]. Companion cats in NZ are defined as common domestic cats that reside with humans and depend on them for their welfare [2]. Most owners choose to have a companion cat because of the friendship, love, and affection they offer. The negative effect companion cats have on the environment is, however, often overlooked. Free-roaming cats are considered to have several negative environmental impacts [3, 4], with one of the most notable being wildlife predation [5-10]. In addition, free-roaming cats are implicated in other less understood ecological effects, such as disease transmission and behavioral changes (e.g., breeding behavior, parental care, and stress induction) [11]. The spread of *toxoplasmosis gondii* by cats is of particular concern [4] as it poses a risk to humans [12] and marine mammals such as endangered Māui and Hector's dolphins in NZ [13]. The welfare of free-roaming cats is also at risk due to their increased likelihood of injury and death [4, 5, 9]. As such, there is a clear need for cat owners to manage their cat's roaming behavior, for both environmental and cat welfare reasons.

Cat management behaviors

Cat containment is one of several cat management behaviors owners can engage in to reduce their cat's environmental impact and the likelihood of roaming-related accidents [14, 15]. It includes a variety of behaviors and ranges from keeping a cat indoors at all times and providing controlled outdoor access (e.g., limiting a cat to an escape-proof fenced yard, an enclosure / run, or walks on a harness and lead), to keeping a cat indoors for a period of time (e.g., overnight) [4, 14]. Cat containment is, however, a novel practice in NZ, with research finding that less than 14% of NZ owners kept their cat indoors or on their property at all times and only 14-29% inside overnight [14, 16]. Interestingly, the performance of cat containment has been found to vary significantly by country and region [16, 17]. According

to Hall et al., [16], approximately two thirds of owners in NZ (67%) and the United Kingdom (UK; 64%) let their cat roam freely, while owners in Australia (80-92%) and the United States (80-93%) commonly performed some form of cat containment (indoors overnight, controlled outdoor access, or indoors at all times), and in Japan most kept their cat indoors (75%).

Behavior change interventions

To improve cat management practices in NZ, evidence-based interventions may be necessary to change cat owner's behavior. Interestingly, McLeod et al., [18] who conducted an audit of the cat management interventions employed by organizations internationally, found that many effective behavior change techniques were under-utilized. They found that most organizations relied on inappropriate techniques, such as logical, evidenced-based messaging, and discussed that the application of behavior change theory would allow for more effective interventions to be developed. It has been suggested that theory-based behavior change interventions to address animal management issues should follow these four key steps: 1) select a human behavior to target which has the greatest potential to address the issue, 2) determine the barriers and drivers underlying non-participation in the prioritized behavior, 3) develop interventions that directly address the barriers and drivers to increase engagement in the behavior, and 4) evaluate the effectiveness of the intervention in promoting behavior change [19, 20]. For the issue of free-roaming companion cats in NZ, Linklater et al., [14] conducted the first step by applying the McKenzie-Mohr behavior prioritization framework [21]. They considered the beneficial impact of various behaviors, their likelihood of adoption among owners, the proportion of owners already performing them, and veterinarians' opinions. Of the behaviors investigated they found that cat containment was the most effective behavior to target and promote among NZ cat owners (particularly keeping cats on the property always and overnight). With cat containment

supported as the best behavior to target, the next step is to understand the barriers and drivers of cat containment, which can then help to design interventions to increase participation in this behavior in NZ [19, 20].

The COM-B model of behavior and audience segmentation

A broad range of domains have applied the COM-B model [22] to understand the barriers and drivers of behavior related to health [23-29], pro-environmental behavior [30-32], agriculture [33, 34], and invasive animal management [35-37]. According to COM-B, behavior is the result of three main factors: capability, opportunity, and motivation (COM) [22]. Capability is a person's psychological and physical capacity to perform a behavior. It includes factors such as skills, awareness, knowledge, and confidence in one's ability to perform a behavior. Opportunity is comprised of physical and social opportunity and are external factors that enable a behavior to be performed. Physical opportunity includes having the necessary time, environmental and financial circumstances, and access to resources required to perform a behavior. Social Opportunity includes social norms and influences that make performance of a behavior more or less likely. Motivation includes internal factors that direct a behavior. It comprises reflective motivation (the conscious beliefs, attitudes, and goals which inform decision making) and automatic motivation (the emotional associations, habits, and impulses that subtly direct behavior). COM-B can be an overarching framework that integrates behavioral determinants specified by different behavioral theories relevant to invasive animal management (e.g., theory of planned behavior, health belief model, protection motivation theory) [38]. An additional strength of this model is that it sits within the Behavior Change Wheel (BCW). The BCW links each COM component with appropriate interventions to address it and thereby modify behavior [22]. For instance, according to the BCW, if non-participation in a desired behavior is the result of low physical capability,

techniques such as training or enablement are recommended to increase physical capability and thereby change behavior.

Audience segmentation analysis can also be conducted to develop an in-depth understanding of the barriers and drivers of cat containment [19, 20]. Audience segmentation allows for the determination of whether a population is a single audience with the same set of factors impacting behavior, or whether multiple segments exist, each with a different set of factors influencing behavior [19, 20, 39]. While this is an approach commonly used by marketers, it has also been utilized in the context of climate change communications [40-42], pro-environmental behavior [43, 44], health [45], and invasive animal management [35-37, 46]. Previous research on invasive animal management has demonstrated that populations are not homogenous, with multiple segments identified that varied in their level of support for a desired behavior [36, 37, 46]. Identifying unique audiences can help to decide which segments to target for behavior change and whether interventions should be tailored to suit the characteristics of each audience [19, 20].

Identifying the barriers and drivers of cat containment behavior

Preliminary research has sought to identify the barriers and drivers of owners' cat containment behavior and to determine whether there are distinct audiences of cat owners. In the NZ context, MacDonald et al., [47] applied the theory of planned behavior (TPB) to understand the influences on owners' intentions to keep their cat inside at night. They found that 31% of owners contained their cat at night and that the most influential predictors of night-containment intentions were attitudes, particularly the belief that night-containment is beneficial for the cat. They also found that household members and veterinarians influenced through injunctive norms night-containment intentions and that other cat owners' lack of engagement in night-containment predicted non-night-containment intentions. These findings suggest that attitudes and norms may be drivers of night-containment behavior.

International research offers additional insight into the influences on cat containment behavior and the types of audience segments that may exist in NZ. Firstly, Tan et al., [17] found that perceived benefits and risk of roaming were related to allowing cats to free roam. In addition, demographics and cat characteristics were related to cat containment behavior, which was also demonstrated by an international study [48]. In the AU context van Eeden et al., [49] applied the TPB and found that 83% of cat owners surveyed in the state of Victoria performed cat containment (30% indoors overnight, 53% controlled outdoor access or indoors at all times). The most important predictors of this behavior included perceived ability to perform cat containment and beliefs (concerns about cat safety, cats' right to roam, and cat predation behavior). In the UK, Crowley et al., [46] identified five distinct cat owner segments based on their beliefs, attitudes, and emotional reactions related to their cat's roaming and predation behavior. Their findings suggested that a key motivator to perform cat containment for some groups was cat welfare, while for others it was reducing predation of wildlife. Lastly, McLeod et al., [35] found that owners surveyed in AU can be segmented into four distinct audiences based on their cat containment behavior, and that the majority contained their cat at all times or overnight (65%). Owners who *always* contained their cat (33%) were more confident in their ability to perform containment, had more positive attitudes towards the benefit of cat containment, and were more likely to contain their cat if it was compulsory (injunctive social norm), compared to those who only had a *night curfew* for their cat (32%), *sporadically* (29%), or *never* (6%) contained their cat.

Overall, previous research [17, 35, 46, 47, 49] supports that COM factors such as psychological capability, social norms, beliefs, attitudes, and emotional reactions predict cat containment behavior, and that distinct audiences of cat owners exist which differ in COM factors. However, these studies have several limitations which must be noted. Firstly, in NZ MacDonald et al., [47] only investigated night containment intentions, and so the influences

on NZ cat owners' performance of the full scope of cat containment behaviors remain unknown. Secondly, research has primarily focused on assessing the impact of motivation and social norms on cat containment [17, 46, 47, 49], with only one study having assessed a range of COM factors [35]. Thirdly, aside from one study [17], previous study populations were limited to either one [35, 47, 49] or two localities [46], meaning previous findings may not be generalizable to wider populations of cat owners. Finally, only one study [35] assessed whether distinct segments of cat owners differed in their behavior and this study mainly highlighted differences between participants and non-participants in cat containment. This was likely due to by the high participation in cat containment (65%) that was found [35]. As such, how non-participants in cat containment may differ from one another remains unclear.

While preliminary research internationally has assessed the barriers and drivers of cat containment and conducted audience segmentation analysis, further research is still required in the NZ context for several reasons. Firstly, the majority of NZ owners do not participate in cat containment [14, 16], and so it is not yet known how COM-B profiles differ in a large population of non-participants. Secondly, Hall et al., [16] in an international survey of cat owners found that attitudes towards various cat management practices and perception of the issues posed by companion cats to wildlife varied significantly by country. In AU, for instance, the majority of owners believed there is a need for cat management legislation and that cats should be kept inside at night, compared with less than 40% of NZ owners and only around 20% of UK owners. Furthermore, nearly two-thirds of AU owners perceived companion cats killing wildlife to be a serious issue, compared to less than half of NZ owners and only around 10% of UK owners. According to the COM-B model [22] these differing beliefs suggest that NZ owners may be less motivated to contain their cats than AU owners, but more so than UK owners. Therefore, it can be concluded that despite the cultural and environmental similarities between NZ, AU, and the UK, there are likely different factors

influencing the adoption of cat containment behavior and different segments of cat owners in each country.

The Current Study

Previous research has identified barriers and drivers that influence owners' adoption of cat containment and that distinct audiences of owners may exist with unique COM-B profiles [17, 35, 46, 47, 49]. However, despite this body of research, two key questions remain unanswered. Firstly, what is the comprehensive set of COM factors that act as barriers and drivers of NZ owners' participation in cat containment, and secondly can NZ owners be segmented into unique audiences based on their COM profiles, that differ significantly in their performance of cat containment. This study sought to address these gaps in the literature using data from a quantitative cross-sectional online survey of NZ cat owners [50].

Consistent with the COM-B model of behavior [22] and previous research in invasive animal management [17, 19, 35-38, 46, 47, 49], the following hypotheses were made: 1) cat owners with increased capability, opportunity, and motivation to contain will have greater cat containment intentions and behavior, and 2) at least three segments of cat owners will exist in NZ which differ significantly in the set of COM factors predicting their cat containment behavior. The findings from this study will inform the design of empirically grounded behavioral interventions that address the causes of non-participation in cat containment for different audiences of cat owners in NZ, which will thereby improve the management of companion cats.

Method

Participants

Participants were recruited using a Lucid Marketplace online sample [51] (between (February 3, 2022 and February 10, 2022) and comprised 395 cat owners residing in NZ,

aged 18 years and over. Reviewing previous research that assessed the relationship between barriers and drivers of cat containment and behavior and intentions demonstrated that the smallest expected effect size for this study was $f^2 = .20$ [47, 52, 53]. Furthermore, a previous study that segmented cat owners into audience groups based on their cat containment beliefs found a five segment solution [46]. As such a power analysis using G*Power [54], with a power level of .90 and an α level of .05, was conducted based on an expected five segment solution. The power analysis indicated at least 390 participants were required for adequate statistical power.

In the sample 254 participants identified as female (64%), 137 as male (35%) and four as other (1%) [50]. The mean year of birth was 1974 (*SD* = 17.18, range = 1931 to 2004), meaning participants were approximately 48 years old on average (*Median* = 48). Most participants identified as NZ European (76%), followed by Māori (10%), and other (14%). The majority had an undergraduate qualification or greater (38%), followed by a secondary school qualification (29%). Compared to the 2018 NZ census, females and NZ European were over-represented, and participants had an older median age and were more educated (NZ 2018 census: 51% female; median age of 37; 25% with an undergraduate qualification or greater; 70% NZ European [55]). However, according to Companion Animals New Zealand [1], this is consistent with NZ cat owners. They found that cat ownership rates were highest in females (43%) and adults aged 35-64 years (43%-53%) compared with New Zealanders overall (41%). Moreover, cat ownership was significantly higher among NZ Europeans (46%) compared to other ethnic groups such as Māori (36%).

Procedure and materials

An online survey was conducted in February 2022 using the Qualtrics online survey platform [56]. The study received blanket ethics approval from the Human Ethics Committee of the University of Canterbury. Participants' informed written consent was obtained at the

beginning of the questionnaire. Demographics information was collected first, then definitions of ‘free-roaming cats’, ‘cat containment’, ‘cat enclosure’, and ‘cat escape-proof fence’ were provided, and after that cat containment intentions and behaviors were assessed. Finally, items pertaining to COM to perform cat containment were completed. Participants who did not pass the security checks (e.g., bot and duplicate response detection; n = 23), did not currently own a cat (n = 508), were born after 2004 (n = 1), and completed the questionnaire in less than 4 minutes or failed the three attention check items (e.g., “to show that you are paying attention, we ask you to select ‘agree’”; n = 112) were screened out. The questionnaire is shown in S1 Appendix.

Measures

The questionnaire measured cat containment intentions and behavior, and COM to perform cat containment.

Cat containment intentions and behavior

Cat containment was assessed with two subscales, Containment Intentions and Containment Behavior. Containment Intentions were measured with seven items asking participants how often in the next six months they expected to do the following behaviors: allow cat to roam freely, keep cat indoors, keep cat indoors overnight, confine cat to an escape-proof fenced yard when outside, confine cat to an enclosure when outside, walk cat on a harness and lead when outside, and fully supervise cat when outside. After reverse scoring the free-roaming item, the items were averaged into a single scale which demonstrated adequate internal consistency (Cronbach’s α = .84). Containment Behavior was assessed with seven items asking participants how often they currently did the following behaviors: allow cat to roam freely, keep cat indoors, keep cat indoors overnight, confine cat to an escape-proof fenced yard when outside, confine cat to an enclosure when outside, walk cat on a harness and lead when outside, and fully supervise cat when outside [35]. After reverse

scoring the free-roaming item, the items were averaged into a single scale, which demonstrated adequate internal consistency (Cronbach's α = .83). All responses were assessed on a five-point Likert scale (1 = never, 2 = rarely, 3 = sometimes, 4 = often, 5 = always).

COM variables

To assess cat owners' capability, opportunity, and motivation related to containment, seven subscales were developed with 51 items (S2 Table). The items were drawn or adapted from previous qualitative and quantitative research on barriers and drivers of cat containment [35, 46, 52, 53, 57]. To ensure all subdimensions of COM were comprehensively assessed, additional items were also drawn from related invasive species management research or developed based on behavioral theory [20, 22, 37, 38].

Capability was assessed with a single subscale, Capability to Contain, which comprised nine items measuring cat owners' physical capability, awareness and knowledge of the issue, memory / attention capacity, and behavioral regulation towards cat containment. All responses were assessed on a five-point Likert scale (1 = strongly disagree, 2 = disagree, 3 = neither agree nor disagree, 4 = agree, 5 = strongly agree). Five items were reverse scored so that higher scores on all items reflected greater capability to contain. Following reverse scoring, the items were averaged to create a single scale, which demonstrated adequate internal consistency (Cronbach's α = .70).

Opportunity was assessed with two subscales, Physical and Social Opportunity to Contain. Physical Opportunity to Contain was measured with eight items that assessed time, resource availability, and environmental context relevant to cat containment. These responses were assessed on a five-point Likert scale (1 = strongly agree, 2 = agree, 3 = neither agree nor disagree, 4 = disagree, 5 = strongly disagree), with higher scores reflecting greater physical opportunity to contain. The scores for the items were averaged to create a single scale, which

demonstrated adequate internal consistency (Cronbach's α = .81). Social Opportunity to Contain was measured with four items. The items assessed interpersonal influences on cat containment (e.g., veterinarians, other cat owners). Responses were assessed on a five-point Likert scale (1 = strongly oppose, 2 = oppose, 3 = neutral, 4 = support, 5 = strongly support), with higher scores indicating greater social opportunity to contain. The scores for the items were averaged to create a single scale, which demonstrated adequate internal consistency (Cronbach's α = .86).

Motivation was assessed with four subscales; Concern About Roaming, Containment is Beneficial for Cat Beliefs, Pro-Containment Beliefs, and Automatic Motivation to Contain. Concern About Roaming was measured using 11 items, assessing perceived levels of concern about potential issues related to cats roaming freely, such as injury or death on the road and killing wildlife. Responses were assessed on a five-point Likert scale (1 = unconcerned, 2 = slightly concerned, 3 = somewhat concerned, 4 = very concerned, 5 = extremely concerned), with higher scores indicating greater concern about consequences of roaming. The scores for the items were then averaged to create a single scale which demonstrated high internal consistency (Cronbach's α = .95). Containment is Beneficial for Cat Beliefs was measured with four items, assessing the perceived impact of containment on a cat's quality of life. Responses were assessed on a five-point Likert scale (1 = very harmful, 2 = harmful, 3 = neither beneficial nor beneficial, 4 = beneficial, 5 = very beneficial) with higher scores indicating greater perceived benefit of containment for cats. The scores for the items were averaged to create a single scale which demonstrated adequate internal consistency (Cronbach's α = .80). Pro-Containment Beliefs were measured with 13 items, assessing beliefs and attitudes towards containment and roaming, perceived effort of containment, and social role identity. All responses were assessed on a five-point Likert scale (1 = strongly disagree, 2 = disagree, 3 = neither agree nor disagree, 4 = agree, 5 = strongly agree). Nine

items were reverse scored so that higher scores on all items represented stronger pro-containment beliefs. The scores for the items were averaged to create a single scale which demonstrated high internal consistency (Cronbach's α = .90). Automatic Motivation to Contain was measured with two items, assessing emotions towards containment and containment habits on a five-point Likert scale (1 = strongly agree, 2 = agree, 3 = neither agree nor disagree, 4 = disagree, 5 = strongly disagree), with higher scores indicating stronger automatic motivation to contain. The scores for the items were averaged to create a single scale, which demonstrated adequate internal consistency (Cronbach's α = .81).

Statistical analyses

Descriptive analysis & bivariate correlations were conducted to assess relationships among all variables, followed by multiple regression analyses to determine the degree to which COM variables predicted Containment Intentions and Behavior. Latent profile analysis (LPA) was then used to classify participants into homogenous subgroups based on their scores on COM variables. Bayesian Information Criterion (BIC), Akaike's Information Criterion (AIC) and Entropy were used to assess the relative model fit of the profile solutions and the Lo-Mendell-Rubin (LMR) test was used for model comparison. Lower AIC and BIC values indicate better model fit whilst lower Entropy values indicate greater uncertainty in the model [58-61]. LMR is a significant test that compares the likelihood ratio of a model with one which has less profile groups [61, 62]. Multivariate analysis of variance (MANOVA) was then conducted to validate the profiles and determine what proportion of variance in the cat containment behavioral variables was explained by the subgroups. Follow up analyses of variance (ANOVAs) were then used to test for significant differences in the two cat containment behavioral variables between the segments. Mplus 7.0 was used to conduct the LPA [63] and SPSS was used for all other analyses [64].

Results

Descriptive statistics & bivariate correlations for all study variables

A summary of the full sample means, standard deviations, and intercorrelations for all variables are shown in Table 1. All COM variables had moderate to large significant associations with Containment Intentions and Behavior ($r > .30$) [65]. As expected, increases in levels of the COM variables were all associated with increases in cat owners' cat containment intentions and behavior.

Predicting cat containment behavior with the COM variables

Multiple regression was used to investigate the extent to which COM factors predicted cat containment intentions and behavior. In the first analysis, the factors Capability to Contain, Physical Opportunity to Contain, Social Opportunity to Contain, Concern About Roaming, Containment is Beneficial for Cat Beliefs, Pro-Containment Beliefs, and Automatic Motivation to Contain were used to predict Containment Intentions, and in the second analysis these factors were used to predict Containment Behavior. All variables were simultaneously entered into the models.

The results from the multiple regression analyses for Containment Intentions is summarized in Table 2 and Containment Behavior in Table 3. Capability to Contain, Concern About Roaming, Containment is Beneficial for Cat Beliefs, and Automatic Motivation to Contain were all significant predictors of Containment Intentions and Containment Behavior. These results demonstrate that cat owners intended to perform and performed cat containment to a greater extent when they; 1) had the psychological and physical capability to contain their cat, 2) were concerned about the negative consequences of roaming, 3) believed containment is beneficial for a cat, and 4) had automatic motivation to perform cat containment. While Physical Opportunity to Contain, Social Opportunity to Contain, and

Pro-Containment Beliefs were not significant predictors of Containment Intentions and Behavior, they were all individually significantly associated with Containment Intentions and Behavior (Table 1). Overall, the models explained 57% of the variance in Containment Intentions and 56% of the variance in Containment Behavior. Of the variance explained in Containment Intentions, Containment is Beneficial for Cat Beliefs and Concern About Roaming each uniquely explained 3%, Automatic Motivation to Contain 2%, and Capability to Contain 1%. Of the variance explained in Containment Behavior, Containment is Beneficial for Cat Beliefs uniquely explained 4%, Concern About Roaming 3%, Automatic Motivation to Contain 2%, and Capability to Contain 1%.

Table 1. Descriptive statistics and correlations for all variables

Variable	*M*	*SD*	1	2	3	4	5	6	7	8	9
1. Containment Behavior	1.85	0.75	—								
2. Containment Intentions	1.87	0.80	.95*<br>[.92, .96]	—							
3. Capability to Contain	3.15	0.65	.49*<br>[.41, .56]	.50*<br>[.42, .57]	—						
4. Physical Opportunity to Contain	2.87	0.92	.33*<br>[.24, .42]	.34*<br>[.26, .42]	.61*<br>[.53, .67]	—					
5. Social Opportunity to Contain	2.69	0.85	.48*<br>[.39, .56]	.50*<br>[.42, .57]	.30*<br>[.19, .39]	.22*<br>[.12, .32]	—				
6. Concern About Roaming Beliefs	2.78	1.06	.49*<br>[.41, .57]	.50*<br>[.42, .58]	.30*<br>[.20, .40]	.18*<br>[.07, .28]	.38*<br>[.28, .47]	—			
7. Containment is Beneficial for Cat Beliefs	2.59	0.79	.66*<br>[.60, .71]	.66*<br>[.60, .71]	.45*<br>[.36, .53]	.32*<br>[.22, .41]	.61*<br>[.54, .67]	.48*<br>[.39, .56]	—		
8. Pro-Containment Beliefs	2.59	0.72	.66*<br>[.60, .73]	.68*<br>[.62, .74]	.60*<br>[.51, .66]	.44*<br>[.35, .53]	.64*<br>[.57, .71]	.49*<br>[.40. .58]	.73*<br>[.68, .77]	—	
9. Automatic Motivation to Contain	2.07	1.03	.63*<br>[.55, .70]	.64*<br>[.57, .70]	.55*<br>[.46, .62]	.41*<br>[.32, .50]	.53*<br>[.43, .61]	.32*<br>[.22, .41]	.63*<br>[.55, .70]	.80*<br>[.74, .84]	—

Note: $N = 395$. Pearson's correlation coefficient, $r = .30$ specifies a medium effect size and $r = .50$ a large effect size [65]. Values in square brackets indicate bias corrected and accelerated bootstrap 95% confidence interval per correlation.

All variables range from a minimum 1 to maximum 5.

* $p < .001$ (2-tailed).

Table 2. Predicting containment intentions from capability, opportunity, and motivation factors of containment

Predictors	B	95% CI for B		SE B	*β*	sr^2
		LB	UB			
Constant	-.47	-.77	-.17	.15	-	-
Capability to Contain	.13*	.01	.25	.06	-.11	.01
Physical Opportunity to Contain	-.00	-.07	.07	.04	-.00	.00
Social Opportunity to Contain	.03	-.05	.11	.04	.03	.00
Concern About Roaming	.14**	.09	.20	.30	.20	.03
Containment is Beneficial for Cat Beliefs	.27**	.16	.37	.05	.26	.03
Pro-Containment Beliefs	.15	-.01	.30	.08	.13	.00
Automatic Motivation to Contain	.18**	.10	.27	.04	.24	.02

Notes: $N = 395$. B = unstandardized beta coefficients. CI = confidence interval. LB = lower bound. UB = upper bound. β = standardized beta coefficients. sr^2 = squared semi-partial correlation coefficient (the proportion of variance the predictor uniquely explained in the dependent variable over and above the other predictors). All predictors ranged from a minimum 1 to maximum 5.

$R^2 = .57$. Adjusted $R^2 = .57$.

* $p < .050$, ** $p < .001$.

Table 3. Predicting containment behavior from capability, opportunity, and motivation factors of containment

Predictors	B	95% CI for B		SE B	*β*	sr^2
		LB	UB			
Constant	-.29	-.57	-.00	.15	-	-
Capability to Contain	.13*	.02	.24	.06	-.01	.01
Physical Opportunity to Contain	-.01	-.08	.06	.04	-.01	.00
Social Opportunity to Contain	.01	-.08	.09	.04	.01	.00
Concern About Roaming	.14**	.08	.19	.30	.20	.03
Containment is Beneficial for Cat Beliefs	.29**	.19	.39	.05	.30	.04
Pro-Containment Beliefs	.09	-.06	.24	.08	.08	.00
Automatic Motivation to Contain	.18**	.10	.27	.04	.25	.02

Notes: $N = 395$. B = unstandardized beta coefficients. CI = confidence interval. LB = lower bound. UB = upper bound. β = standardized beta coefficients. sr^2 = squared semi-partial correlation coefficient (the proportion of variance the predictor uniquely explained in the dependent variable over and above the other predictors). All predictors ranged from a minimum 1 to maximum 5.
$R^2 = .56$. Adjusted $R^2 = .55$.
* $p < .050$, ** $p < .001$.

Segmentation of cat owners based on COM variables

LPA was conducted to determine whether there are multiple unique segments of cat owners, that differ in their COM to perform cat containment. The results identified four different cat owner profiles based on the COM variables. Table 4 shows a summary of the LPA model fit indices pertaining to two to five profile solutions. The five-profile solution had the lowest AIC and BIC values, indicating it had the best model fit, however, LMR indicated that a five-profile solution did not significantly better fit the data than a four-profile solution. In addition, with an Entropy value greater than .80, the four-profile solution is supported to

have minimal uncertainty in its profile classification of individuals (according to Celeux and Soromenho, and Tein et al., as cited in Ferguson et al., [61]). Therefore, the four-profile solution was retained for interpretation. The segments were labelled; *engaged, receptive, ambivalent,* and *opposed.* The deviation of each segment from the sample mean for the COM variables is shown in Fig 1.

Table 4. Model fit indices for two to five cat owner profile solutions

Profile Solution	AIC	BIC	Entropy	LMR	*p*
2	6171.47	6259.00	.91	770.62	.070
3	5823.78	5943.14	.85	356.24	.380
4	5659.76	5810.96	.87	176.33	.008
5	5621.56	5804.53	.84	53.15	.550

Notes: AIC = Akaike's Information Criterion. BIC = Bayesian Information Criteria. LMR = Lo-Mendel-Rubin likelihood ratio test. Lower values of AIC and BIC indicate better model fit, and low entropy values indicate more uncertainty in a model's classification of individuals [58-61]. LMR is a significant test that compares the likelihood ratio of a model with one that has less profile groups [62].

Fig. 1. Standardized means of the capability, opportunity, and motivation factors across the four cat owner profiles. *Notes:* Engaged, n = 22. Receptive, n = 68. Ambivalent, n = 188. Opposed, n = 117. Standardized means reflect the segment's deviation from the sample mean on the variables.

The smallest segment *engaged* (*n* = 22, 6%), had the highest levels of all COM variables relative to the other segments. Members of this segment had the strongest Automatic Motivation to Contain, Pro-Containment Beliefs, and Capability to Contain. The *receptive* segment (*n* = 68, 17%) had the second highest levels of all COM variables. They

had moderate Automatic Motivation to Contain, Pro-Containment Beliefs, Containment is Beneficial for Cat Beliefs, and Social Opportunity to Contain. Their Capability to Contain and Physical Opportunity to Contain, while still above average, were however relatively lower than the other COM factors. The largest segment, *ambivalent* (n = 188, 48%), had overall average levels of the COM variables. Their Social Opportunity to Contain, Concern About Roaming, and Containment is Beneficial for Cat Beliefs were slightly above average relative to others in the sample. In addition, their Capability to Contain, Physical Opportunity to Contain, and Automatic Motivation to Contain were slightly below average. The *opposed* segment (n = 117, 30%) had the lowest overall levels of the COM variables. They had the lowest Pro-Containment Beliefs, Containment is Beneficial for Cat Beliefs, and Social Opportunity to Contain.

Demographic characteristics of cat owner profiles

In terms of demographic characteristics, the *receptive* segment had the highest proportion of females (68%) and the *engaged* and *opposed* segments had the highest proportion of NZ Europeans (both 86%). The *engaged* segment had the highest proportion of those living in urban localities (urban, suburban, rural residential; 95%) and the *ambivalent* and *opposed* segments had the largest proportion of those with a medium to large garden at their residence (90% and 88% respectively). One-way ANOVAs found that the segments did not differ significantly in their year of birth, $F(3, 391) = 1.98$, $p = .116$ or education level, $F(3, 391) = 1.56$, $p = .199$). Unstandardized segment descriptive statistics for all study variables are shown in S3 Table.

Validation of cat owner segments

The relationship between membership in the four cat owner segments and the cat containment behavioral variables were then examined using MANOVA to confirm if segment membership predicted cat containment. Two follow up ANOVAs with post-hoc tests

were then used to test for significant differences in Containment Intentions and Behavior between the segments.

The MANOVA demonstrated that the four cat owner profiles explained 31% of the variance in the cat containment behavioral variables, $\eta^2 = .31$, $V = .47$, $F(6, 780) = 59.13$, $p < .001$. Two follow up one-way ANOVAs were then conducted to determine the group effect for each behavioral variable. Levene's test for equality of variance was significant for Containment Intentions ($p < .001$) and Containment Behavior ($p < .001$). Given the heterogeneity of variances, the more robust Welch's *F* test was used for the univariate significance tests [66]. A significant effect of group on levels of Containment Intentions, $\eta^2 = .52$, $F(3, 79.55) = 107.28$, $p < .001$, and on levels of Containment Behavior $\eta^2 = .49$, $F(3, 80.05) = 89.07$, $p < .001$ was found.

To determine which groups differed significantly, post-hoc comparisons using the Games-Howell test, which is considered the most accurate when population variances are unequal, were conducted (according to Toothaker, as cited in Field [66]). All segments were found to differ significantly from each other in their Containment Intentions ($p \leq .005$) and Containment Behavior ($p \leq .003$). As shown in Fig 2, the *engaged* segment had the highest levels of both Containment Intentions ($M = 3.35$, $SD = .71$) and Containment Behavior ($M = 3.27$, $SD = .73$), followed by *receptive* (Containment Intentions $M = 2.70$, $SD = .87$; Containment Behavior $M = 2.58$, $SD = .77$), *ambivalent* (Containment Intentions $M = 1.73$, $SD = .50$; Containment Behavior $M = 1.72$; $SD = .50$), and *opposed* (Containment Intentions $M = 1.34$, $SD = .33$; Containment Behavior $M = 1.38$; $SD = .38$). These results support that distinct audiences of cat owners exist among surveyed owners with their cat containment intentions and behavior being determined by different COM profiles.

Fig. 2. Means of the intentions and behavior variables for the four cat owner profiles. Notes: Engaged, *n* = 22. Receptive, *n* = 68. Ambivalent, *n* = 188. Opposed, *n* = 117. The error bars represent 95% confidence intervals. All variables ranged from a minimum 1 to maximum 5.

Discussion

This study sought to identify the barriers and drivers of cat containment for NZ cat owners. Firstly, it was investigated whether cat owners with greater capability, opportunity, and motivation (COM) to perform cat containment were more likely to contain their cat. Secondly, this study sought to determine if unique segments of cat owners with different COM profiles and cat containment behaviors exist in NZ. This thereby extended the literature by rigorously assessing both cat containment intentions and behavior of NZ cat owners, with consideration given to the wide variety of behaviors that can comprise cat containment practices (e.g., night-containment, cat-enclosures, always inside). Furthermore, by applying the COM-B framework [22], this study identified additional predictors of cat containment and determined whether NZ cat owners, who are mostly non-participants in cat containment, can be segmented into unique audiences based on their COM profiles.

Our study found that cat containment was rarely intended to be performed, and rarely performed by NZ cat owners. Engagement in cat containment was found to be positively associated with all COM factors and it was uniquely predicted by capability and motivational factors. In addition, this study identified four audiences of cat owners who differed in the COM factors predicting their cat containment intentions and behavior. These findings are explored below and practical implications, recommendations for future research, and acknowledgements of the limitations of this study are provided.

What predicts cat owners' participation in cat containment?

As hypothesized, higher levels of COM to perform cat containment predicted cat containment intentions and behavior. All capability, opportunity, and motivational factors assessed in this study were positively associated with cat containment intentions and behavior. The factors that uniquely predicted intentions and behavior were Capability to Contain, Concern About Roaming, Containment is Beneficial for Cat Beliefs, and Automatic Motivation to Contain. Although Physical and Social Opportunity to Contain and Pro-Containment Beliefs were not significant predictors over-and-above the other factors, they were each independently associated with intentions and behavior. As such, these findings demonstrate that cat containment was performed to a significantly greater extent when cat owners had the capability to perform containment (e.g., skills, memory, knowledge, awareness, and behavioral regulation), had physical and social opportunity enabling containment (e.g., time, environmental circumstances, and interpersonal influences), were concerned about the negative consequences of roaming (e.g., cat being injured or killing wildlife), believed that containment has a beneficial impact on a cat's quality of life, had pro-containment beliefs, and had greater automatic motivation to contain.

This study extends previous findings by undertaking to the best of our knowledge, the most comprehensive assessment of cat owners' COM to perform cat containment to date. Furthermore, this study demonstrated that a range of additional factors are statistically reliable predictors of cat containment intentions and behavior. This is consistent with the COM-B model of behavior which states that behavior is determined by a person's capability, opportunity, and motivation [22]. Finally, this study builds upon previous findings which demonstrated that psychological capability, beliefs, attitudes, and social influences were predictors of cat containment [17, 35, 46, 47, 49, 53].

Audience segmentation of cat owners based on COM

As hypothesized, audience segmentation analysis demonstrated that NZ cat owners could be segmented into four subgroups, which differed significantly in the set of COM factors predicting their behavior. By supporting that four different audiences of cat owners may exist in NZ, tailored interventions can now be designed to address the underlying causes of non-participation for each audience identified and decisions can be made about who should be targeted for behavior change [19, 20]. The four cat owner segments were labelled; *engaged*, *receptive*, *ambivalent*, and *opposed*. *Engaged* cat owners (6%) were characterized by the highest COM factors and the highest participation in cat containment, followed by *receptive* (17%), and *ambivalent* cat owners (48%). *Opposed* cat owners (30%) were characterized by the lowest COM factors and the lowest participation in cat containment.

These findings have some similarities with other research, but also some key differences. Crowley et al., [46] identified five cat owner segments based on their beliefs, attitudes, and emotional reactions, however, did not consider situational and contextual factors. Furthermore, they did not evidence whether membership in these segments predicted behavior. While McLeod et al., [35] also identified four cat-owner segments that differed significantly in their behavior, the largest segment identified were those who *always* contained their cat, while in the current study those who *engaged* in cat containment were the smallest segment. This suggests that NZ cat owners are mostly non-participants in cat containment compared to those in AU, which is consistent with previous research [16]. NZ owners therefore appear to face greater barriers and less drivers of cat containment. In addition, McLeod et al., [35] found that the main COM differences were between the segment who *always* contained their cat and the segments that did not perform containment (*night curfew*, *sporadic*, and *never*). Those who *always* contained had stronger perceived behavioral control, beliefs about the benefit of containment to cats, and social normative

influences than the other groups. The current study, however, found that all identified segments differed from each other on a broad set of COM factors. These differences may be due to the more comprehensive set of COM factors assessed in this study, leading to a more nuanced understanding of cat owners surveyed. Additionally, it may suggest that cross-cultural variations exist, with NZ cat owners, who are mainly non-participants in cat containment, being more heterogeneous on a larger range of COM factors than AU cat owners. Reasons for this may include the success of cat containment campaigns that have taken place in AU since the 1990s [14, 35, 49], the additional wildlife risks to cats in AU (e.g., venomous snakes; [57]), and the more extensive cat management regulations in AU compared to NZ [67].

Practical implications

The findings from this study have several implications for practitioners seeking to increase participation in cat containment, such as government agencies and wildlife protection and animal welfare organizations. The identification of different audiences of cat owners suggests that it may be necessary to tailor interventions to each segment to promote behavior change. Furthermore, targeting *receptive* and *ambivalent* cat owners, who represented around two thirds of those surveyed and exhibited fewer barriers to change, could provide the best opportunity to exponentially increase cat containment practices in NZ.

The behavior change wheel (BCW) [22] can be applied to identify appropriate behavior change techniques for each segment [19, 20]. The BCW is a framework which links capability, opportunity, and motivational factors with strategies to address them [22]. For those *receptive* to cat containment, factors that need to be addressed were Capability to Contain, Physical Opportunity to Contain, and Concern About Roaming. According to the BCW [22], Capability to Contain could be increased through *training*, for instance, by offering workshops for cat owners on how to build their own cat enclosure. Physical

Opportunity to Contain could be increased through *environmental restructuring* (e.g., improving the availability of cat containment products such as cat escape-proof fence systems). To increase Concern About Roaming, *education* could be used to inform cat owners about the prevalence of roaming-related accidents to cats [57]. The same techniques could also be applied to *ambivalent* cat owners, who also have low Capability to Contain, Physical Opportunity to Contain, and Concern About Roaming. However, consideration should also be given to improving their low Automatic Motivation to Contain, for instance through *modeling*. Influencers who perform cat containment in social media campaigns could act as role models to create positive associations with cat containment for *ambivalent* cat owners (e.g., showing off their 'catio' to their followers).

Whilst practitioners may prioritize targeting *receptive* and *ambivalent* cat owners, some consideration should also be given to *engaged* and *opposed* cat owners. *Opposed* cat owners could be shifted to become more *ambivalent* or even *receptive* to cat containment by using *persuasion* to address their lack of Pro-Containment Beliefs [18, 22]. This could involve using credible messengers, such as veterinarians, to communicate the benefits of enrichment for contained cats (e.g., providing food puzzles) and to persuade them that contained cats can be healthy and happy [20, 22, 68]. Finally, the techniques outlined could also be used to maintain the high participation in cat containment in *engaged* cat owners.

Limitations & future research

Limitations of this research include the use of self-report measures of behavior and a cross-sectional study design. While self-report measures have a number of benefits, they can have a recall and social desirability bias, as has been found in the assessment of lifestyle behaviors [69, 70], and thus lead to over- or under-reporting of behavior. In addition, the cross-sectional design of this study does not allow for strong directional causal inferences between COM factors and behavior to be determined [71]. Finally, while participants'

demographic backgrounds appeared broadly consistent with NZ cat owners [1], the present study relied on an online panel sample which may not be generalizable to the wider population of NZ cat owners.

Future research using a longitudinal research design with intensive measurement of behavior could be conducted to better understand the direction of the relationship between the COM factors and behavior, and to prevent recall bias. Additionally, research should explore whether cat owner segments differ in their demographic backgrounds (e.g., gender, locality, dwelling type) and the characteristics of the owned cat (e.g., breed, sex, health status, behavioral issues). Finally, research should design interventions using a tool such as the BCW [22] to directly address the set of COM factors found to predict the cat containment behavior of the different cat owner audiences, in congruence with the behavior change process outlined by Hine et al., [20] and McLeod et al., [19]. Experimental designs with long-term follow ups can be used to assess the efficacy and effectiveness of interventions in promoting enduring behavior change in cat owners.

Conclusion

This study demonstrated that a sample of NZ cat owners are largely non-participants in cat containment, with various capability, opportunity, and motivational factors predicting their cat containment intentions and behavior. This study extends our understanding of cat containment behavior by supporting the influence situational and contextual factors have on whether NZ cat owners contain their cats or not. Audience segmentation analysis revealed that cat-owners surveyed were not a homogenous group, with four segments identified that have unique sets of COM factors predicting cat containment. More than three quarters were either *ambivalent* or *opposed* to containment, with only a minority being *engaged* or *receptive* towards it. Interventions seeking to promote cat containment among NZ cat owners

should target those *receptive* and *ambivalent* towards cat containment to have the greatest overall impact on the issue of free-roaming companion cats.

Acknowledgements

We thank all the participants in this study for the time and input they provided. A special thanks as well to Andreas K. Jaeger for his support and proof-reading.

References

1. Companion Animals New Zealand. Companion animals in New Zealand 2020. 2020.
2. National Animal Welfare Advisory Committee. Code of Welfare: Companion Cats. Wellington: Ministry for Primary Industries (MPI), 2018.
3. Kikillus KH, Chambers GK, Farnworth MJ, Hare KM. Research challenges and conservation implications for urban cat management in New Zealand. Pacific Conservation Biology. 2016;23(1):15-24. doi: https://doi.org/10.1071/PC16022
4. Tan SML, Stellato AC, Niel L. Uncontrolled outdoor access for cats: an assessment of risks and benefits. Animals. 2020;10(2):258. PubMed PMID: doi:10.3390/ani10020258.
5. Bruce SJ, Zito S, Gates MC, Aguilar G, Walker JK, Goldwater N, et al. Predation and risk behaviors of free-roaming owned cats in Auckland, New Zealand via the use of animal-borne cameras. Frontiers in Veterinary Science. 2019;6:205-. doi: 10.3389/fvets.2019.00205.
6. van Heezik Y, Smyth A, Adams A, Gordon J. Do domestic cats impose an unsustainable harvest on urban bird populations? Biological Conservation. 2010;143(1):121-30. doi: https://doi.org/10.1016/j.biocon.2009.09.013.
7. Flux JEC. Seventeen years of predation by one suburban cat in New Zealand. New Zealand Journal of Zoology. 2007;34(4):289-96. doi: 10.1080/03014220709510087.

8. Gordon J, Matthaei C, Van Heezik Y. Belled collars reduce catch of domestic cats in New Zealand by half. Wildlife research. 2010;37(5):372-8. doi: https://doi.org/10.1071/WR09127.

9. Loyd K, Hernandez S, Abernathy K, Shock B, Marshall G. Risk behaviours exhibited by free- roaming cats in a suburban US town. Veterinary Record. 2013;173(12):295-. doi: https://doi.org/10.1136/vr.101222

10. Woods M, McDonald RA, Harris S. Predation of wildlife by domestic cats Felis catus in Great Britain. Mammal review. 2003;33(2):174-88. doi: https://doi.org/https://doi.org/10.1046/j.1365-2907.2003.00017.x

11. Medina FM, Bonnaud E, Vidal E, Nogales M. Underlying impacts of invasive cats on islands: not only a question of predation. Biodiversity and Conservation. 2014;23:327-42. doi: https://doi.org/10.1007/s10531-013-0603-4.

12. Lepczyk CA, Lohr CA, Duffy DC. A review of cat behavior in relation to disease risk and management options. Applied Animal Behaviour Science. 2015;173:29-39. doi: https://doi.org/10.1016/j.applanim.2015.07.002.

13. Department of Conservation. Toxoplasmosis and Hector's and Māui dolphin n.d. Available from: https://www.doc.govt.nz/nature/pests-and-threats/diseases/toxoplasmosis-and-hectors-and-maui-dolphin/.

14. Linklater WL, Farnworth MJ, van Heezik Y, Stafford KJ, MacDonald EA. Prioritizing cat- owner behaviors for a campaign to reduce wildlife depredation. Conservation Science and Practice. 2019;1(5):e29. doi: https://doi.org/10.1111/csp2.29

15. Cecchetti M, Crowley SL, Wilson-Aggarwal J, Nelli L, McDonald RA. Spatial behavior of domestic cats and the effects of outdoor access restrictions and

interventions to reduce predation of wildlife. Conservation Science and Practice. 2022;4(2):e597. doi: https://doi.org/10.1111/csp2.597.

16. Hall CM, Adams NA, Bradley SJ, Bryant KA, Davis AA, Dickman CR, et al. Community attitudes and practices of urban residents regarding predation by pet cats on wildlife: an international comparison. PLoS ONE. 2016;11(4):e0151962. doi: 10.1371/journal.pone.0151962.

17. Tan SML, Jajou S, Stellato AC, Niel L. Perspectives of Canadian and American cat owners on provision of uncontrolled outdoor access for owned domestic cats. Frontiers in Veterinary Science. 2021;8. doi: 10.3389/fvets.2021.742245.

18. McLeod LJ, Driver AB, Bengsen AJ, Hine DW. Refining Online Communication Strategies for Domestic Cat Management. Anthrozoös. 2017;30(4):635-49. doi: 10.1080/08927936.2017.1370237.

19. McLeod LJ, Hine DW, Driver AB. Change the humans first: Principles for improving the management of free-roaming cats. Animals. 2019;9(8):555. doi: https://doi.org/10.3390/ani9080555

20. Hine DW, McLeod LJ, Driver AB. Designing behaviour change interventions for invasive animal control: a practical guide. Centre for Invasive Species Solution: Canberra, Australia. 2019.

21. McKenzie-Mohr D. Fostering sustainable behavior: An introduction to community-based social marketing: New Society Publishers; 2011.

22. Michie S, van Stralen MM, West R. The behaviour change wheel: A new method for characterising and designing behaviour change interventions. Implementation Science. 2011;6(1):42. doi: 10.1186/1748-5908-6-42.

23. Flannery C, McHugh S, Anaba AE, Clifford E, O'Riordan M, Kenny LC, et al. Enablers and barriers to physical activity in overweight and obese pregnant women:

an analysis informed by the theoretical domains framework and COM-B model. BMC Pregnancy and Childbirth. 2018;18(1):1-13. doi: https://doi.org/10.1186/s12884-018-1816-z.

24. McDonagh LK, Saunders JM, Cassell J, Curtis T, Bastaki H, Hartney T, et al. Application of the COM-B model to barriers and facilitators to chlamydia testing in general practice for young people and primary care practitioners: a systematic review. Implementation Science. 2018;13(1):1-19. doi: https://doi.org/10.1186/s13012-018-0821-y.

25. Barker F, Atkins L, de Lusignan S. Applying the COM-B behaviour model and behaviour change wheel to develop an intervention to improve hearing-aid use in adult auditory rehabilitation. International journal of audiology. 2016;55(sup3):S90-S8. doi: 10.3109/14992027.2015.1120894.

26. Williams KN, Thompson LM, Sakas Z, Hengstermann M, Quinn A, Díaz-Artiga A, et al. Designing a comprehensive behaviour change intervention to promote and monitor exclusive use of liquefied petroleum gas stoves for the Household Air Pollution Intervention Network (HAPIN) trial. BMJ open. 2020;10(9):e037761. doi: 10.1136/bmjopen-2020-037761.

27. Thompson LM, Diaz-Artiga A, Weinstein JR, Handley MA. Designing a behavioral intervention using the COM-B model and the theoretical domains framework to promote gas stove use in rural Guatemala: a formative research study. BMC Public Health. 2018;18(1):1-17. doi: https://doi.org/10.1186/s12889-018-5138-x.

28. West R, Michie S, Rubin GJ, Amlôt R. Applying principles of behaviour change to reduce SARS-CoV-2 transmission. Nature Human Behaviour. 2020;4(5):451-9. doi: 10.1038/s41562-020-0887-9.

29. Herber OR, Atkins L, Störk S, Wilm S. Enhancing self-care adherence in patients with heart failure: a study protocol for developing a theory-based behaviour change intervention using the COM-B behaviour model (ACHIEVE study). BMJ open. 2018;8(9):e025907. doi: http://dx.doi.org/10.1136/bmjopen-2018-025907.

30. Webb J, Moxon S. A study protocol to understand urban rewilding behaviour in relation to adaptations to private gardens. Cities & Health. 2021:1-9. doi: https://doi.org/10.1080/23748834.2021.1893047.

31. Sundaraja CS, Hine DW, Lykins AD. Palm oil: Understanding barriers to sustainable consumption. PLoS ONE. 2021;16(8):e0254897. doi: 10.1371/journal.pone.0254897.

32. Allison AL, Lorencatto F, Michie S, Miodownik M. Barriers and enablers to food waste recycling: a mixed methods study amongst UK citizens. International journal of environmental research and public health. 2022;19(5):2729.

33. Kropf B, Schmid E, Schönhart M, Mitter H. Exploring farmers' behavior toward individual and collective measures of Western Corn Rootworm control–A case study in south-east Austria. Journal of Environmental Management. 2020;264:110431. doi: https://doi.org/10.1016/j.jenvman.2020.110431.

34. Prosser N, Green M, Ferguson E, Tildesley M, Hill E, Keeling M, et al. Cattle farmer psychosocial profiles and their association with control strategies for bovine viral diarrhea. Journal of Dairy Science. 2022;105(4):3559-73. doi: 10.3168/jds.2021-21386.

35. McLeod LJ, Hine DW, Bengsen AJ. Born to roam? Surveying cat owners in Tasmania, Australia, to identify the drivers and barriers to cat containment. Preventive Veterinary Medicine. 2015;122(3):339-44. doi: https://doi.org/10.1016/j.prevetmed.2015.11.007

36. McLeod LJ, Hine DW. Using audience segmentation to understand nonparticipation in invasive mammal management in Australia. Environmental Management. 2019;64(2):213-29. doi: https://doi.org/10.1007/s00267-019-01176-5

37. Hine DW, McLeod LJ, Please PM. Understanding why peri-urban residents do not report wild dog impacts: an audience segmentation approach. Human Dimensions of Wildlife. 2020;25(4):355-71. doi: 10.1080/10871209.2020.1735579.

38. McLeod LJ, Hine DW, Please PM, Driver AB. Applying behavioral theories to invasive animal management: towards an integrated framework. Journal of Environmental Management. 2015;161:63-71.

39. Slater MD. Theory and method in health audience segmentation. Journal of Health Communication. 1996;1(3):267-84. doi: https://doi.org/10.1080/108107396128059.

40. Maibach EW, Leiserowitz A, Roser-Renouf C, Mertz CK. Identifying Like-Minded Audiences for Global Warming Public Engagement Campaigns: An Audience Segmentation Analysis and Tool Development. PLoS ONE. 2011;6(3):e17571. doi: 10.1371/journal.pone.0017571.

41. Hine DW, Phillips WJ, Cooksey R, Reser JP, Nunn P, Marks ADG, et al. Preaching to different choirs: How to motivate dismissive, uncommitted, and alarmed audiences to adapt to climate change? Global Environmental Change. 2016;36:1-11. doi: https://doi.org/10.1016/j.gloenvcha.2015.11.002.

42. Hine DW, Reser JP, Morrison M, Phillips WJ, Nunn P, Cooksey R. Audience segmentation and climate change communication: conceptual and methodological considerations. Wiley Interdisciplinary Reviews: Climate change. 2014;5(4):441-59. doi: 10.1002/wcc.279.

43. Malek L, Umberger WJ, Goddard E. Committed vs. uncommitted meat eaters: Understanding willingness to change protein consumption. Appetite. 2019;138:115-26. doi: https://doi.org/10.1016/j.appet.2019.03.024.

44. Morgan MI, Hine DW, Bhullar N, Loi NM. Landholder adoption of low emission agricultural practices: A profiling approach. Journal of Environmental Psychology. 2015;41:35-44. doi: https://doi.org/10.1016/j.jenvp.2014.11.004.

45. Bloem S, Stalpers J, Groenland EA, van Montfort K, van Raaij WF, de Rooij K. Segmentation of health-care consumers: psychological determinants of subjective health and other person-related variables. BMC Health Services Research. 2020;20(1):1-12. doi: https://doi.org/10.1186/s12913-020-05560-4.

46. Crowley SL, Cecchetti M, McDonald RA. Diverse perspectives of cat owners indicate barriers to and opportunities for managing cat predation of wildlife. Frontiers in Ecology and the Environment. 2020;18(10):544-9. doi: https://doi.org/10.1002/fee.2254.

47. MacDonald E, Milfont T, Gavin M. What drives cat-owner behaviour? First steps towards limiting domestic-cat impacts on native wildlife. Wildlife Research. 2015;42(3):257-65. doi: https://doi.org/10.1071/WR14164

48. Foreman-Worsley R, Finka LR, Ward SJ, Farnworth MJ. Indoors or outdoors? An international exploration of owner demographics and decision making associated with lifestyle of pet cats. Animals. 2021;11(2):253. doi: https://doi.org/10.3390/ani11020253.

49. van Eeden LM, Hames F, Faulkner R, Geschke A, Squires ZE, McLeod EM. Putting the cat before the wildlife: Exploring cat owners' beliefs about cat containment as predictors of owner behavior. Conservation Science and Practice. 2021;3(10):e502. doi: https://doi.org/10.1111/csp2.502.

50. Chamberlain SAE, Mcleod LJ, Hine DW. Barriers and drivers of cat containment behavior for New Zealand cat owners. 2023. Mendeley Data, V2. doi: 10.17632/hytxh9bm8c.1.

51. Lucid. Lucid Marketplace. 2022.

52. McLeod LJ, Evans D, Jones B, Paterson M, Zito S. Understanding the relationship between intention and cat containment behaviour: a case study of kitten and cat adopters from RSPCA Queensland (Royal Society for the Prevention of Cruelty to Animals). Animals (Basel). 2020;10(7). doi: 10.3390/anil0071214.

53. Toukhsati SR, Young E, Bennett PC, Coleman GJ. Wandering cats: attitudes and behaviors towards cat containment in Australia. Anthrozoös. 2012;25(1):61-74. doi: 10.2752/175303712X13240472427195.

54. Faul F, Erdfelder E, Lang A-G, Buchner A. G*Power 3: A flexible statistical power analysis program for the social, behavioral, and biomedical sciences. Behavior Research Methods. 2007;39(2):175-91. doi: 10.3758/BF03193146.

55. Stats NZ. Ethnic group summaries reveal New Zealand's multicultural make-up. 2022, September 3.

56. Qualtrics. Survey Software. 2022.

57. Elliott A, Howell TJ, McLeod EM, Bennett PC. Perceptions of responsible cat ownership behaviors among a convenience sample of Australians. Animals (Basel). 2019;9(9):703. doi: 10.3390/ani9090703.

58. Schwarz G. Estimating the dimension of a model. The annals of statistics. 1978:461-4.

59. Ramaswamy V, DeSarbo WS, Reibstein DJ, Robinson WT. An empirical pooling approach for estimating marketing mix elasticities with PIMS data. Marketing Science. 1993;12(1):103-24. doi: https://doi.org/10.1287/mksc.12.1.103.

60. Akaike H. Information Theory and an Extension of the Maximum Likelihood Principle. In: Parzen E, Tanabe K, Kitagawa G, editors. Selected Papers of Hirotugu Akaike: Springer 1998. p. 199-213.

61. Ferguson SL, G. Moore EW, Hull DM. Finding latent groups in observed data: A primer on latent profile analysis in Mplus for applied researchers. International Journal of Behavioral Development. 2020;44(5):458-68. doi: http://dx.doi.org/10.1177/0165025419881721.

62. Lo Y, Mendell NR, Rubin DB. Testing the number of components in a normal mixture. Biometrika. 2001;88(3):767-78. doi: https://doi.org/10.1093/biomet/88.3.767.

63. Muthén L, Muthén B. MPlus. 7.0 ed: Muthén & Muthén, Los Angeles, CA; 2022.

64. IBM. IBM SPSS statistics for Apple macOS 26 ed: IBM Corp, Armonk, NY; 2022.

65. Cohen J. Statistical power analysis for the behavioral sciences: Routledge; 2013.

66. Field A. Discovering Statistics using IBM SPSS Statistic (5th ed.): Sage Publications; 2018.

67. Sumner CL, Walker JK, Dale AR. The implications of policies on the welfare of free-roaming cats in New Zealand. Animals. 2022;12(3):237. doi: https://doi.org/10.3390/ani12030237.

68. Dantas LM, Delgado MM, Johnson I, Buffington CT. Food puzzles for cats: feeding for physical and emotional wellbeing. Journal of Feline Medicine and Surgery. 2016;18(9):723-32.

69. Schoeller DA. Limitations in the assessment of dietary energy intake by self-report. Metabolism. 1995;44:18-22. doi: https://doi.org/10.1016/0026-0495(95)90204-X.

70. Prince SA, Adamo KB, Hamel ME, Hardt J, Gorber SC, Tremblay M. A comparison of direct versus self-report measures for assessing physical activity in adults: a

systematic review. International Journal of Behavioral Nutrition and Physical Activity. 2008;5(1):56. doi: 10.1186/1479-5868-5-56.

71. Wang X, Cheng Z. Cross-sectional studies: strengths, weaknesses, and recommendations. Chest. 2020;158(1, Supplement):S65-S71. doi: https://doi.org/10.1016/j.chest.2020.03.012.

Supporting information

S1 Appendix. Cat containment questionnaire

S2 Table. Capability, opportunity, and motivation variables and survey items

S3 Table. Summary of descriptive statistics for the four cat owner profiles for the capability, opportunity, and motivations (COM) factors and demographics

Figure 1

Click here to access/download;Figure;Fig1.tif

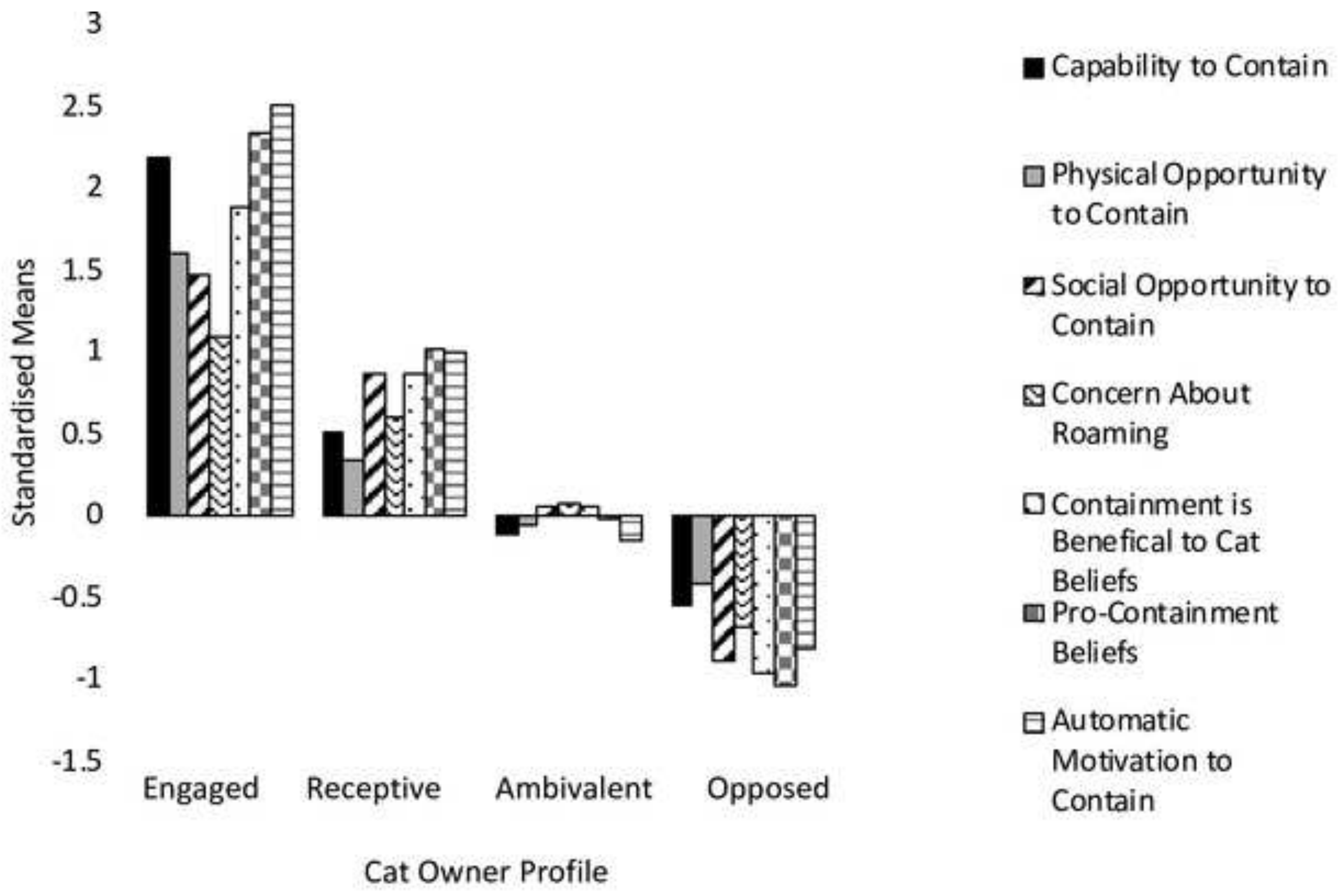

Fig. 1. Standardized means of the capability, opportunity, and motivation factors across the four cat owner profiles. *Notes:* Engaged, n = 22. Receptive, n = 68. Ambivalent, n = 188. Opposed, n = 117. Standardized means reflect the segment's deviation from the sample mean on the variables.

Figure 2

Click here to access/download;Figure;Fig2.tif

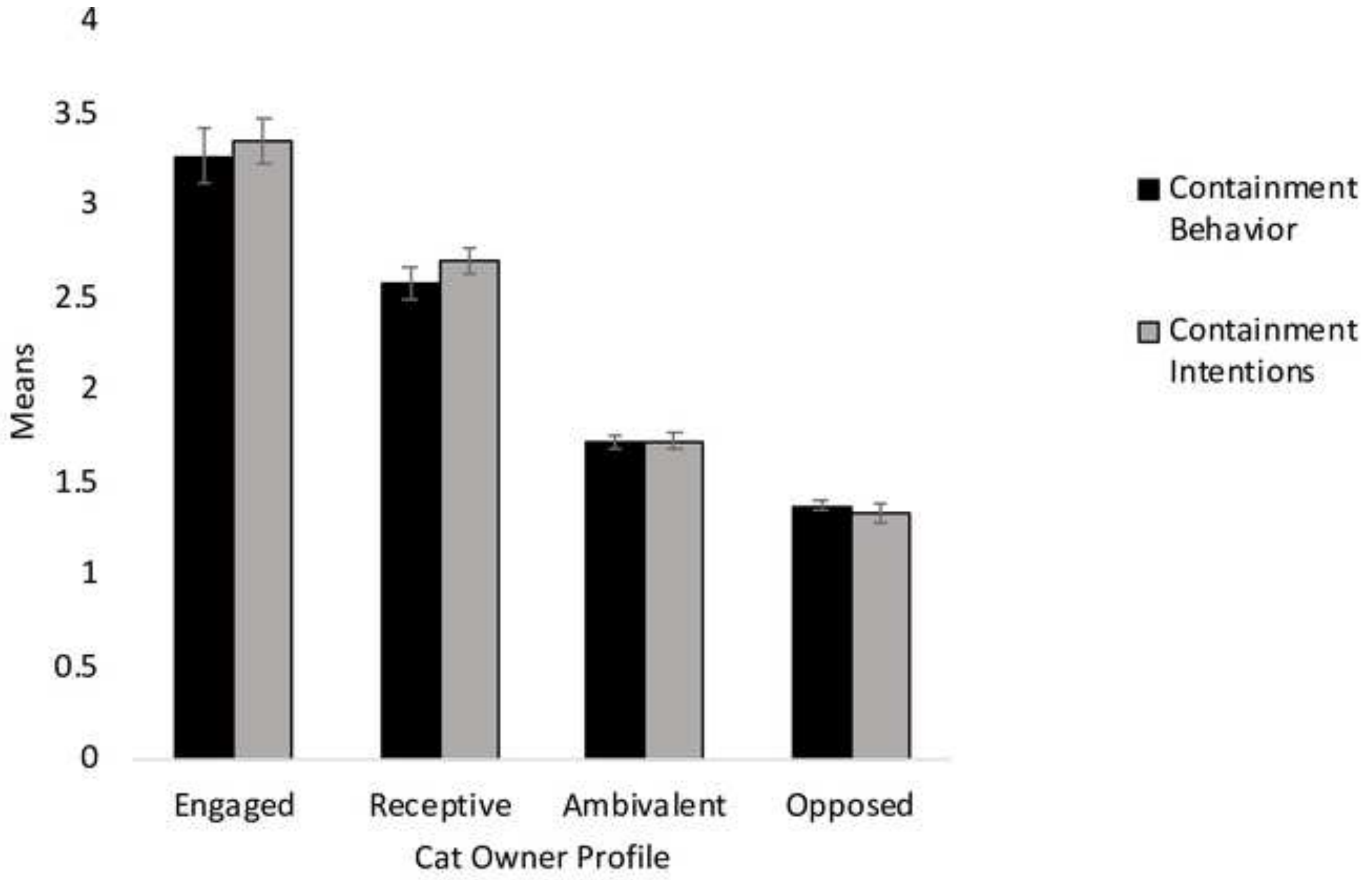

Fig. 2. Means of the intentions and behavior variables for the four cat owner profiles.

Notes: Engaged, $n = 22$. Receptive, $n = 68$. Ambivalent, $n = 188$. Opposed, $n = 117$. The error bars represent 95% confidence intervals. All variables ranged from a minimum 1 to maximum 5.

Click here to access/download
Supporting Information
S1_Appendix.pdf

Click here to access/download
Supporting Information
S2_Table.pdf

Supporting Information 3

Click here to access/download
Supporting Information
S3_Table.pdf
